# Alu-miRNA interactions modulate transcript isoform diversity in stress response and reveal signatures of positive selection

**DOI:** 10.1038/srep32348

**Published:** 2016-09-02

**Authors:** Rajesh Pandey, Aniket Bhattacharya, Vivek Bhardwaj, Vineet Jha, Amit K. Mandal, Mitali Mukerji

**Affiliations:** 1 CSIR Ayurgenomics Unit - TRISUTRA, CSIR- Institute of Genomics and Integrative Biology (IGIB), Sukhdev Vihar, Mathura Road, New Delhi-110025, INDIA; 2 Genomics and Molecular Medicine Unit, CSIR- IGIB, Sukhdev Vihar, Mathura Road, New Delhi-110025, INDIA; 3 Academy of Scientific and Innovative Research (AcSIR), New Delhi, INDIA; 4 Persistent LABS, Persistent Systems Ltd., Pune-411004, Maharashtra, INDIA; 5 GN Ramachandran Knowledge Centre for Genome Informatics, CSIR-IGIB, Sukhdev Vihar, Mathura Road, New Delhi-110025, INDIA

## Abstract

Primate-specific Alus harbor different regulatory features, including miRNA targets. In this study, we provide evidence for miRNA-mediated modulation of transcript isoform levels during heat-shock response through exaptation of Alu-miRNA sites in mature mRNA. We performed genome-wide expression profiling coupled with functional validation of miRNA target sites within exonized Alus, and analyzed conservation of these targets across primates. We observed that two miRNAs (miR-15a-3p and miR-302d-3p) elevated in stress response, target *RAD1, GTSE1, NR2C1, FKBP9* and *UBE2I* exclusively within Alu. These genes map onto the p53 regulatory network. Ectopic overexpression of miR-15a-3p downregulates GTSE1 and RAD1 at the protein level and enhances cell survival. This Alu-mediated fine-tuning seems to be unique to humans as evident from the absence of orthologous sites in other primate lineages. We further analyzed signatures of selection on Alu-miRNA targets in the genome, using 1000 Genomes Phase-I data. We found that 198 out of 3177 Alu-exonized genes exhibit signatures of selection within Alu-miRNA sites, with 60 of them containing SNPs supported by multiple evidences (global-F_ST_ > 0.3, pair-wise-F_ST_ > 0.5, Fay-Wu’s H < −20, iHS > 2.0, high ΔDAF) and implicated in p53 network. We propose that by affecting multiple genes, Alu-miRNA interactions have the potential to facilitate population-level adaptations in response to environmental challenges.

The role of Alu elements in shaping the regulatory landscape of the primate transcriptome has recently gained much attention^1^. These ~250 base pair long repeats occur in more than a million copies in the human genome - a feature that complicates their study at the genome-wide scale. However, with the advances in next generation sequencing technologies, their regulatory role at different functional hierarchies, ranging from genomic and epigenetic to transcriptomic and proteomic levels, is increasingly being appreciated^2^,^3^,^4^,^5^,^6^,^7^,^8^,^9^,^10^.

Alus can be transcribed either as free Alu RNA (by their internal Pol III promoter) or as exonized Alus (as a part of the mature mRNA) by Pol II^11^,^12^. Alus also form a major fraction of the antisense transcriptome^13^. Various evidences highlight the role of Alus in regulating cellular homeostasis during stress response^1^,^14^. Alus are responsive to stress and their levels are elevated following heat shock, viral infection and cancer^15^,^16^,^17^. Increased levels of Alu RNA or the impaired activity of DICER1 leads to cytotoxicity in the retinal pigmented epithelial cells, causing age-related macular degeneration^18^. Alu RNA has been shown to act as a transcriptional co-repressor of RNA Pol II and represses transcription of heat shock responsive genes^19^. Presence of cryptic splice sites within Alus potentiates their inclusion into mature mRNAs, preferentially in the 3′UTRs - a process termed as Alu ‘exonization’^20^,^21^,^22^,^23^. Nearly 14% of the human genes can produce an Alu-exonized transcript and ~70% of them are the principal isoforms^12^.

The 3′UTRs of transcripts are known to be the functional hot-spots of miRNA-mediated regulation, which affects mRNA stability and subsequently determines its fate^24^. Earlier genome-wide computational analyses have not only indicated Alus to be the source as well as the target of miRNAs, but have also provided evidence for their co-evolution in the genome^25^,^26^,^27^,^28^. Since genes that can potentially form 3′UTR Alu-exonized transcripts are enriched in nucleotide metabolism and DNA integrity check point pathways, Alu-miRNA interactions could influence these pathways^12^. Recently, it has also been shown that Alus in the 3′UTR of *MDM2* and *MDM4* are targeted by primate-specific miR-661, adding another layer of regulation onto the p53 network^29^. The functionality of Alu-miRNA targets has been demonstrated for a few miRNAs like miR-24, 122 and 1285^30^. Among all the transposons, Alu contains the maximum number of miRNA binding sites, some of which also show signatures of conservation^30^. While the role of miRNAs in heat shock response has been reported in HeLa cells, their involvement in regulation through targets within Alu repeats in the Alu-exonized transcripts has not been studied so far^31^.

As several Alu-mediated events converge onto stress response, we studied the role of Alu-miRNA interaction in a heat shock model of stress. Our study revealed that miRNAs induced in response to heat shock can downregulate Alu-exonized transcript isoforms through presence of targets within Alus. The protein levels of important targets, involved in cell survival pathways, are affected when we perturb the expression of an miRNA targeting exonized Alus. This perturbation affects cellular response to DNA damage and cell proliferation. We studied the tissue-specific expression of these miRNAs and the conservation of the Alu targets in primates, which indicated that these sites might have evolved recently as an adaptation to stress in specific tissues.

Variations in the miRNA seed region have the potential to affect gene regulation through miRNA-mediated gene silencing, and hence participate in adaptive changes to the environment^32^. Therefore, the frequency of these variations can be used as a signature of the importance of miRNA site. Using Fisher statistics (F_ST_) as a measure of population differentiation, an earlier study had shown that the loci with high F_ST_ values tend to be enriched for miRNA sites^33^. Through analysis of statistics calculated from the Phase-I of 1000 Genomes project^34^, we identified that many Alu-miRNA sites show signatures of selection, indicating their role in local adaptation in populations.

## Results

### 3′UTR exonized Alus are targeted by miRNAs induced during heat shock response

#### Genome-wide expression profiling and miRNA target prediction

Genome-wide mRNA (work published earlier from our group^14^) and miRNA expression profiling using same batch (passage number matched) of HeLa cells in response to heat shock, revealed differential expression of 4279 transcripts and 32 miRNAs (22 annotated miRNAs and 10 putative miRNAs, miRPlus) (Supplementary Table S1). Eight out of these 32 differentially expressed miRNAs had their targets within Alu in the 3′UTR of Alu-exonized transcripts. Two of the miRNAs - miR**-**302d-3p and miR**-**15a-3p - exhibited approximately 2.5 fold elevated expression following heat shock in the qPCR validation. We first asked if these two miRNAs have targets in the transcripts that are differentially expressed under identical condition. A consensus of target predictions by miRanda and TargetScan, revealed 398 and 917 genes to be the putative targets for miR-302d-3p and miR-15a-3p, respectively (Supplementary Table S2). Since our miRNAs were found to be upregulated, we limited our query space to only those targets that were downregulated in response to heat shock. miR-302d-3p has its target within 55 genes and miR-15a-3p in 94; eight of them being common targets. In total, nine genes harbored exclusive targets within Alu (referred to as ‘Alu-miRNA targets’ in the subsequent sections), of which miR-15a-3p targets seven genes **-** *NR2C1*, *GTSE1*, *FHL2*, *RAD1*, *FKBP9, CAD* and *SMA4*, and miR**-**302d-3p targets two **-** *ADD1* and *UBE2I* (Supplementary Table S3). miR-15a-3p targets within *CAD* and *SMA4* were present in pseudogenes and hence, were not carried forward for validation. A flow diagram outlining the steps and the filtering criteria used in the experimental work-flow of this paper has been provided in Supplementary Figure S1.

#### Functional validation for the specificity of Alu-miRNA targets

**Figure 1:**
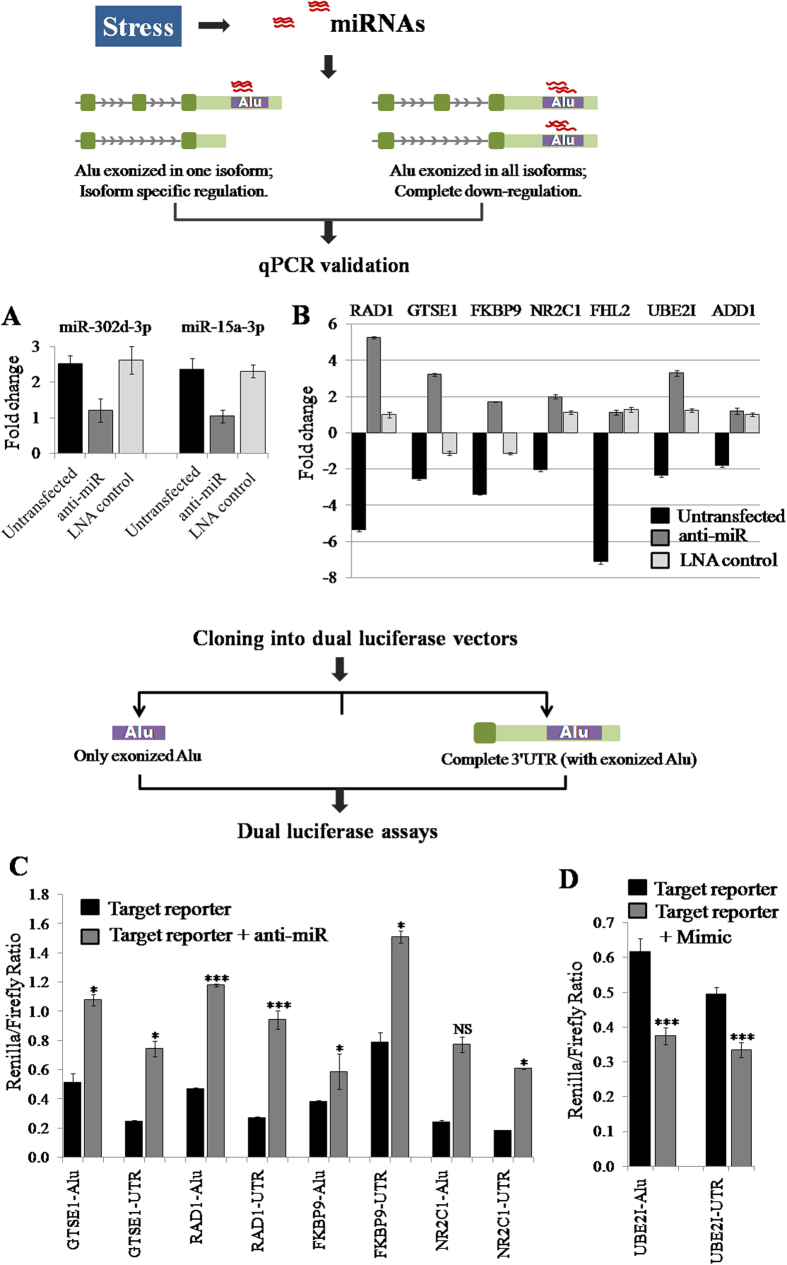
miRNA targets within Alu are functional. (**A**) *Validation of miRNA knockdown.* Downregulation of expression to ~50% compared to the levels of miRNAs during heat shock response in untransfected cells was observed for both miR-15a-3p and miR-302d-3p, validated by qPCR. Experiments were done in triplicates to calculate SD. (**B**) *Anti-miRs rescue the targets of miR-15a-3p and miR-302d-3p during heat shock response.* qPCR validation of seven Alu-miRNA targets show rescue for five of them in the presence of Anti-miR. Anti-miR against miR-15a-3p was used for *NR2C1*, *GTSE1*, *FHL2*, *RAD1* and *FKBP9* whereas miR-302d-3p anti-miR was used for *ADD1* and *UBE2I*. We observed that the transcripts those were originally downregulated during stress (black bar) flip to overexpression in the presence of the respective anti-miR. However, upon scrambled LNA transfection, the effect is non-directional. *FHL2* and *ADD1* did not show reversal of their expression pattern. Experiments were performed in triplicates to calculate SD. (**C**) *Cloning of Alu-miRNA targets confirms the specificity of regulation using anti-miR*. Cloning of the target Alu sequences and the corresponding 3′UTR harboring Alu, downstream of the Renilla luciferase gene was carried out in the psiCHECK2 vector. Ectopic expression of target reporter both in the presence and absence (partial knockdown by anti-miR) of miR-15a-3p was used to confirm target specificity. All the target reporters for Alu-miRNA targets of *NR2C1*, *GTSE1*, *RAD1* and *FKBP9* show upregulation in the presence of miR-15a-3p anti-miR, when compared with the endogenous miRNA levels. The Renilla/firefly luminescence ratio for each experiment was normalized with the ratio of corresponding empty vector control. The empty vector showed non-significant variation among the batches (p = 0.0929, Kruskal-wallis test; Supplementary Table S4). Experiments were performed in duplicates to calculate SD. (**D**) *miR-302d-3p overexpression confirmed UBE2I target.* The target specificity of *UBE2I* was confirmed using an overexpression model of miR-302d-3p wherein target clones showed downregulation in the presence of miR-302d-3p mimic. Expression fold change was calculated with respect to the empty vector, both during the presence and absence of miRNA. The experiment was performed in triplicate to calculate SD. Student’s t-test was performed for statistical significance (^*^p < 0.05, ^**^p < 0.01, ^***^p < 0.005 and NS→non-significant).

Alu-miRNA target specificity was confirmed by knockdown using anti-miRs and the sites were further confirmed using target-specific reporter constructs. HeLa cells were transiently transfected with locked nucleic acid (LNA) modified anti-miRs (at a final concentration of 50 nM) and subsequently treated with heat shock (twenty four hours after transfection), followed by two hours recovery and the levels of miRNAs as well as their target transcripts were queried using qPCR. For these transfection experiments, we maintained both untransfected as well as scrambled LNA transfected controls. The scrambled LNA sequence was of comparable length and synthesized using the same LNA base chemistry as that of the anti-miRs (Exiqon). Anti-miR mediated knockdown showed approximately 50% reduction in the levels of both miR-15a-3p and miR-302d-3p during heat shock response (Fig. 1A). Next, we checked the levels of the target transcripts of these two miRNAs under identical conditions of anti-miR mediated knockdown, followed by heat shock. Each mRNA in each of the states mentioned (untransfected, anti-miR transfected and scrambled LNA transfected) has been checked for expression using *RPL13A* as the reference gene (i.e., the fold change in every case is with respect to *RPL13A*). Upon anti-miR transfection, five out of seven (except *ADD1* and *FHL2)* transcripts harboring Alu-miRNA targets for these miRNAs in their 3′UTR showed upregulation (Fig. 1B). This ‘rescue’, we propose, is mediated by a stoichiometric reduction of the miRNAs induced during heat shock by the specific anti-miRs. We found a flip in the direction of fold change between the two conditions–untransfected, heat shock treated (when the miRNAs were available to bind to their target transcripts) versus anti-miR transfected, heat shock treated (when most of these miRNAs were titrated out by the anti-miRs), but the scrambled treatment showed no such definitive trend.

Subsequently, we cloned both the Alu as well as the complete 3′UTR sequences of the five target genes (*GTSE1, FKBP9, RAD1, NR2C1* and *UBE2I*) into dual luciferase reporter plasmid, psiCHECK2. Expression fold change was calculated with respect to the empty control plasmid, both in the presence (endogenous miRNA level) and absence of miRNA (knockdown using 50 nM anti-miR) (Supplementary Table S4). The ectopic expression of target sites shows significantly higher values, reaching up to 1.5-fold following knockdown of miR-15a-3p (Fig. 1C). However, we did not find any significant upregulation of *UBE2I* after miR-302d-3p knockdown which may be due to its very low endogenous expression. Thus, we used an overexpression model of miR-302d-3p (mimic synthesized by Sigma Aldrich) wherein the mimic was co-transfected along with Alu and 3′UTR clones of *UBE2I*. Indeed, this showed a significant downregulation in both Alu and 3′UTR clones (Fig. 1D). Since Alu elements are known to form secondary structures which might affect regulation, we also checked for possible expression difference between clones for only Alu and corresponding complete 3′UTR. We observed that there is no significant difference in their expression, in response to anti-miR mediated knockdown of miR-15a-3p or overexpression of miR-302d-3p (p = 0.1585 for *RAD1*, 0.0569 for *GTSE1*, 0.0594 for *FKBP9*, 0.2826 for *NR2C1*, 0.0985 for *UBE2I*; Student’s t-test). Thus, it seems that miRNA binding sites within Alu are both accessible for binding as well as can regulate the gene.

#### Functional concordance in Alu-miRNA target genes

**Figure 2:**
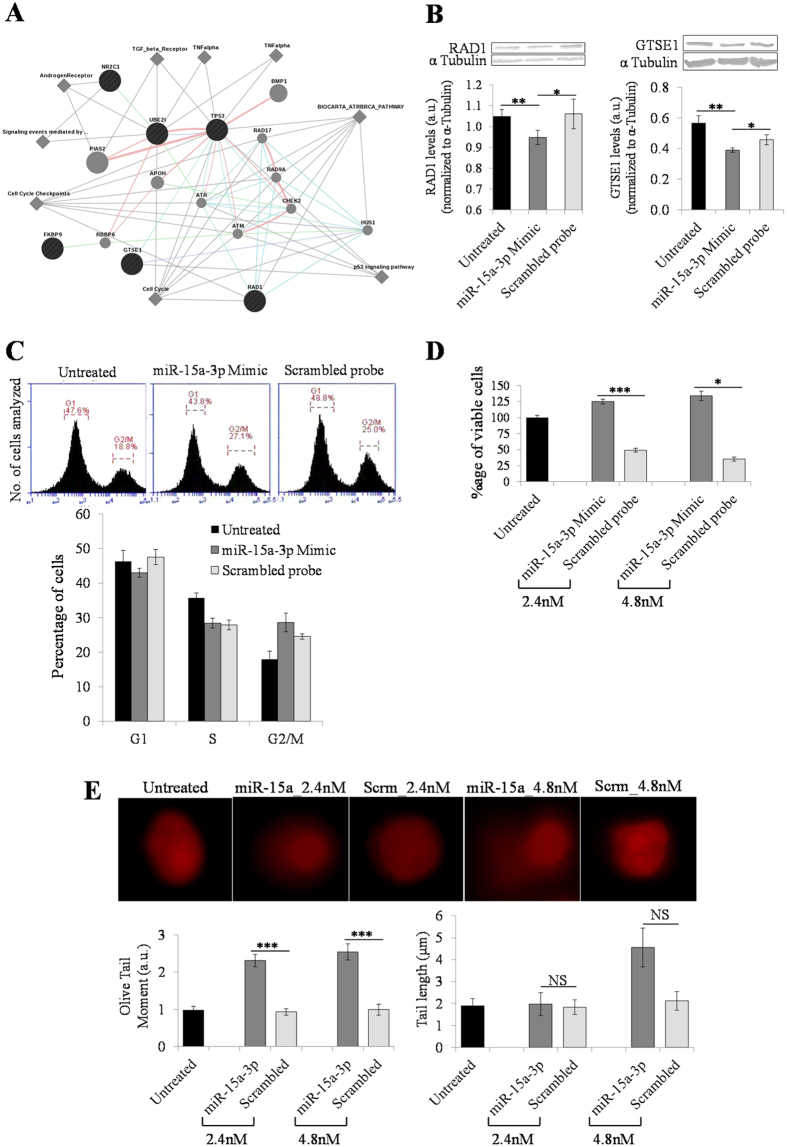
Functional validation of cellular consequences of Alu-miRNA interactions. (**A**) *Network analysis of experimentally validated genes*. Network visualized using GeneMANIA highlights major interacting partners and associated pathways for the genes with Alu-miRNA targets. All the genes interact directly or via an intermediary with p53. The five validated genes (*FKBP9, GTSE1, RAD1, UBE2I* and *NR2C1*) are shown as black nodes, interacting partners in grey and pathways as diamond. (**B**) *miR-15a-3p overexpression downregulates GTSE1 and RAD1.* The ectopic overexpression of 2.4 nM miR-15a-3p shows significant downregulation of both GTSE1 (p = 0.022) and RAD1 (p = 0.042), compared to scrambled, at the protein level. The downregulation is more pronounced for GTSE1 (~30%) compared to that of RAD1 (~10%). Mean (+SD) of two biological replicates are reported here. (**C**) *miR-15a-3p overexpression increases the G2/M cell population.* Treatment with 2.4 nM miR-15a-3p mimic but not with that of scrambled, increases the population of cells in G2/M phase. This facilitates cell division and promotes cell proliferation. The G1 cell population for miR-15a-3p overexpression is comparable to that of the untreated as well as scrambled-treated cells. Mean (+SD) of three biological replicates are reported here. (**D**) *Ectopic expression of miR-15a-3p results in cell proliferation.* Both 2.4 and 4.8 nM treatment with miR-15a-3p mimic show significantly increased viability of cells compared to that of scrambled probe (p = 0.001 and 0.021, respectively). The viability of untreated (no-transfection) cells was taken as 100%. Figure represents the mean (+SD) of three independent experiments. (**E**) *miR-15a-3p overexpression induces DNA damage.* At 2.4 nM treatment with miR-15a-3p mimic, DNA damage is induced; however, it does not result in an extensive fragmentation of genomic DNA (no significant difference in tail length among untreated, mimic and scrambled treated). But at a higher concentration of mimic (4.8 nM), DNA is fragmented to a much greater extent although the index of DNA damage (Olive Tail Moment) remains comparable to that of 2.4 nM treatment. Two biological replicates for each condition were handled in parallel and atleast 30 comets were analyzed for each. Data from both the replicates were pooled to calculate mean (+SD). Student’s t-test was performed to ascertain statistical significance (^*^p < 0.05, ^**^p < 0.01, ^***^p < 0.005 and NS→non-significant).

Using GeneMANIA, we found that all the validated genes harboring Alu-miRNA targets interact directly or via an intermediary with p53, a key molecule regulating cell survival during stress response^35^ (Fig. 2A). For instance, *GTSE1* negatively regulates p53 transactivation function, protein levels and p53-dependent apoptosis whereas *NR2C1* is an orphan nuclear receptor that is regulated by p53^36^,^37^. Cell cycle is regulated by *RAD1* by virtue of its ability to initiate DNA repair, and inhibition of *UBE2I* prevents cell-cycle progression at the G2 or early M phase^38^,^39^.

#### Consequences of miR-15a-3p overexpression at the cellular level

Heat shock is known to evoke a generic pan-cellular stress response which involves multiple players. We wanted to specifically probe the consequences of increased levels of miR-15a-3p in cellular stress response. For this, we ectopically overexpressed miR-15a-3p using its mimic. The purpose of this ectopic expression was to mimic the elevated expression level of this heat shock induced miRNA in a directed set of follow up experiments. For all our overexpression experiments, we have maintained two controls: untransfected as well as scrambled oligo (synthesized by Sigma Aldrich).

miR-15a-3p targets the principal transcript isoforms of *GTSE1* and *RAD1*, proteins that are crucial for cell survival in response to stress. To check whether this post-transcriptional regulation is also reflected at the protein level, we carried out transient transfection assay of miR-15a-3p mimic in HeLa cells. We found a significant downregulation of both the proteins at 2.4 nM ectopic overexpression. The downregulation was more pronounced for GTSE1 (~30%) compared to that of RAD1 (~10%) (Fig. 2B). Subsequent to this finding, we wanted to evaluate the functional consequence of this miRNA-mediated regulation at the cellular level.

GTSE1 is exclusively expressed in the S and G2 phases of the cell cycle and its overexpression retards the progression of the cell to mitosis^36^. Conversely, a decreased level of GTSE1 is expected to promote cell cycle progression. To check this, we sorted miR-15a-3p transfected cells using FACS and quantified the population of cells in different stages of the cell cycle. We observed an increase in the G2/M cell population after 2.4 nM miR-15a-3p transient transfection (Fig. 2C), although the difference from scrambled treatment was not significant (Student’s t-test, p = 0.227). However at a higher dose (4.8 nM), there was a marked increase in the G1 cell population (Supplementary Figure S2). Although, it is known that the introduction of any foreign nucleic acid into the cell tends to block its division by arresting cells at G1, our result highlights the specific role of miR-15a-3p in downregulating GTSE1 which allows cells to cross the G2/M checkpoint. To further ascertain the functional consequence of miR-15a-3p overexpression in increasing G2/M cell population, we checked if it actually results in cell proliferation. Towards this, we performed MTT assay to check the viability/proliferation of cells under similar transfection conditions. We found that both 2.4 and 4.8 nM treatment with miR-15a-3p mimic causes a marked proliferation of cells, unlike that of the scrambled probe which leads to a lot of cell death (Fig. 2D).

RAD1 is a pivotal player in DNA damage response. Being a part of the heterotrimeric complex of RAD9-RAD1-HUS1, it invokes repair mechanism whenever the cell is under genotoxic stress^38^. To find out if the decreased levels of RAD1 leads to compromised DNA repair, we performed alkaline comet assay. At 2.4 nM, we observed that the extent of DNA damage (quantitated by Olive Tail Moment) was significantly higher in the miR-15a-3p mimic treated cells compared to both untreated and scrambled (p = 1.84 × 10^−6^); however, the tail length (an index of the level of DNA fragmentation) was not altered (p = 0.87). In contrast to this, 4.8 nM treatment shows an extensive DNA damage (p = 7.21 × 10^−5^) coupled with longer comet tails (p = 0.08) (Fig. 2E). Thus, it corroborates the higher percentage of cells in the G1 phase which we have observed in the cell cycle progression assay at 4.8 nM (Supplementary Figure S2).

### Alu- miRNA interaction is dynamic in nature

#### Alu containing splice isoforms harbor miRNA targets

We found that two genes - *FKBP9* and *UBE2I* harbor target sites only within the alternate isoforms whereas *GTSE1*, *RAD1* and *NR2C1* have miRNA targets either within the protein coding transcript or in all isoforms (Supplementary Figure S3). All the protein coding as well as alternate isoforms were validated by qPCR for their downregulation in response to heat shock (Fig. 1B).

#### Tissue-specific expression of miRNAs

**Figure 3:**
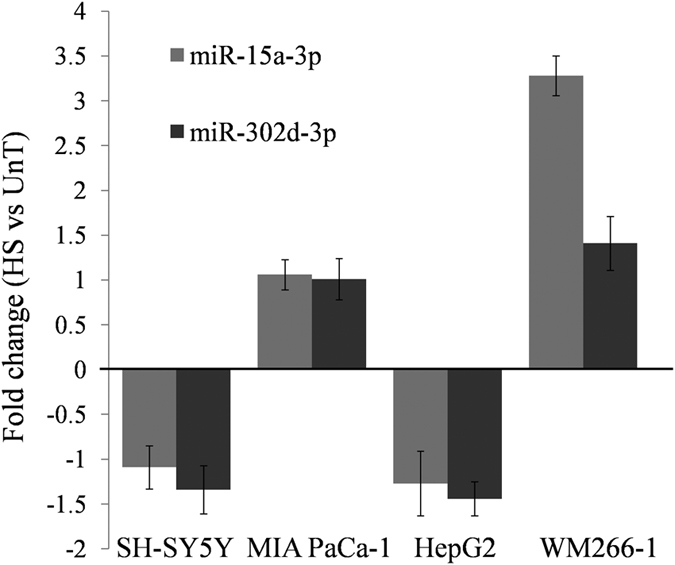
Tissue-specific expression of miRNAs during heat shock response. Both miR-302d-3p and miR-15a-3p were checked for their differential expression in response to heat shock, across four different cell lines belonging to skin (WM266-1), liver (HepG2), pancreas (MIA PaCa-1) and brain (SH-SY5Y). Of these, only the skin cell line (WM266-1) showed more than 3-fold up-regulation for miR-15a-3p, reinforcing the spatio-temporal expression of miRNAs. All the experiments were done in triplicates and represented here with SD.

miRNAs are known to display tissue-specific expression. Thus expanding our observations made in HeLa cells, we checked the expression levels for both these miRNAs, in response to heat shock, across four different cell lines - Brain (SH**-**SY5Y), Pancreas (MIA PaCa**-**1), Liver (HepG2) and Skin (WM266**-**1). We observed that both the miRNAs were expressed at low levels across all the four cell lines, in both treated and untreated conditions. However, in skin cells, miR**-**15a-3p was found to be more than 3-fold upregulated in heat-shock (Fig. 3). All the experiments were carried out using three biological replicates.

### Alu-miRNA target sites show signatures of positive selection

**Figure 4:**
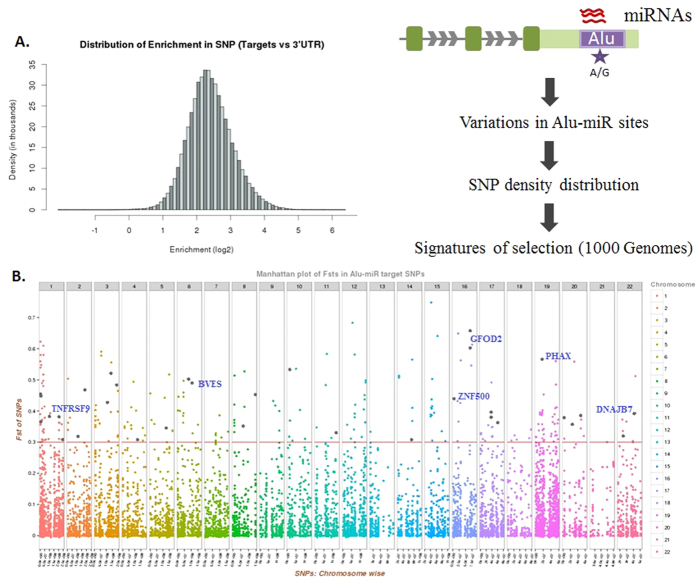
Alu-miRNA targets show signatures of selection. (**A**) *miRNA targets within Alu are enriched for SNPs.* SNPs within the Alu-miRNA targets for all 3′UTR exonized Alus were analyzed using the 1000 Genomes data. The density of SNPs (No. of SNPs per base pair) was calculated for targets within Alu and compared with the corresponding 3′UTR as background. Most of the targets within Alu were found to be 5-6 fold enriched (log2 = 2.5) in the density of SNPs with respect to the corresponding 3′UTR. (**B**) *Various Alu-miRNA targets show signature of selection.* Manhattan plot shows the global F_ST_ values of all the SNPs present within miRNA target sequences of 3′UTR exonized Alus. F_ST_ values were derived from 1000 Genomes populations (YRI, CHB and CEU), obtained using 1000 Genomes selection browser (see Methods). 267 SNPs that show global F_ST_ > 0.3, were analyzed further for their functional importance. The black dots represent those 33 SNPs with an iHS score > 2.0 and derived allele frequency > 0.5 in either of the population/s (Supplementary Table S7). Few important candidates for positive selection in Alu-miRNA target regions like *ZNF500, GFOD2, TNFRSF9, BVES, PHAX* and *DNAJB7*, are also marked.

Since Alus have contributed diverse kinds of regulatory elements in the human genome, we hypothesized that several of these Alu-miRNA target sites, if functional, might exhibit signatures of selection in modern humans. In a genome-wide dataset of 3177 genes that have Alu-exonization^12^, we observed Alu-miRNA targets in 2480 genes, of which 2084 had SNPs within the miRNA target sites. Of the total 40189 SNPs harbored within the 3′UTRs of these 2084 genes, 9139 SNPs were observed within the Alu-miRNA target sites. We found that miRNA target sites (except in three genes) had a higher SNP density in Alu regions when compared to their corresponding 3′UTRs (Fig. 4A). We next analyzed the extent of selective constraint within the Alu-miRNA target sites in 2084 genes using the datasets available in 1000 Genomes selection browser database (Phase I data), which provides multiple indices or signatures of selection using more than 15 population statistics parameters^34^. In the present study, we have focused on five statistical parameters: four frequency based approaches of population differentiation: F_ST_ (Fixation Index), Fay and Wu’s H, Tajima’s D and ΔDAF (difference of Derived Allele Frequency between two populations) and one long range haplotype based measure: iHS (ratio of integrated Haplotype homozygosity Score) in CEU, CHB and YRI populations. We used a filtering approach: first, a global F_ST_ cut-off (>0.3), followed by pair-wise F_ST_ (>0.5, between any population pair) and then Fay and Wu’s H score (<−20 in any of the three populations) (Supplementary Figure S4).

We observed a significant enrichment (p < 2.2 × 10^−16^, Mann-Whitney-Wilcoxon test) of highly differentiated SNPs (global F_ST_ > 0.3) that overlapped with miRNA targets, within Alu-exonized transcripts compared to the non Alu-exonized ones (Supplementary Figure S5). We found a total of 267 SNPs (in 198 genes) with global F_ST_ values >0.3 within Alu-miRNA target sites in exonized transcripts, indicative of their plausible role in population differentiation^40^. To ascertain if there is a bias for such SNPs in these sites, we compared the difference in distribution of all SNPs and only these ‘high’ global F_ST_ SNPs in the Alu and non-Alu regions of the 3′UTR. The following three groups were compared: Group 1 – miRNA targets within all exonized Alus, Group 2 – miRNA targets in non-Alu regions of 3′UTR (canonical miRNA targets) and Group 3 – exonized Alu regions devoid of miRNA targets. We found that Alus with miRNA targets are enriched for SNPs overall as well as for ‘high’ global F_ST_ SNPs compared to the non-Alu region (p < 2.2 × 10^−16^ and p = 0.0008, respectively) within 3′UTR. However, the Alu regions with miRNA targets are not enriched for ‘high’ global F_ST_ SNPs or SNPs overall, compared to Alu regions without miRNA targets (p = 0.089 and p = 0.823, respectively) (Supplementary Figure S5). This is anticipated since Alus have accumulated different types of regulatory elements^8^,^41^, many of which might also be under similar selection constraints. There is also a significant overrepresentation of ‘high’ global F_ST_ and ‘high’ iHS (>2.0) SNPs in the Alu region compared to that of the non-Alu regions of 3′UTR, even after scaling them with the total number of SNPs present in that region (p = 0.02382, Student’s t-test) (Supplementary Figure S6, Supplementary Table S5).

Amongst these 267 SNPs (global F_ST_ > 0.3), we found 144 to be highly differentiated (pair-wise F_ST_ > 0.5) in any of the three population pairs; CEU, CHB and YRI. Since Alus have originated in the primate lineages and are still evolving^8^, we wanted to check if these Alu-miRNA sites encompassing the highly differentiated 144 SNPs exhibit signatures of recent positive selection using the Fay and Wu’s H scores. Using a cut-off of H < −20, we finally arrived at a set of 78 SNPs in 60 genes (Table 1). Out of these, 70 SNPs also had a negative value for Tajima’s D, clearly indicating a deviation from neutrality. The presence of excess high frequency derived SNPs within Alu-miRNA sites, coupled with the above observations are clearly suggestive of recent positive selection. For instance, there is a strong dip in the Fay-Wu’s H score at ~200 kb around the SNP rs10200193 in the 3′UTR of *DUSP19* gene in the CHB population. Another example are the two 3′UTR SNPs (rs10158065, rs11122049) in *NOL9* gene which has quite low H scores in both CEU and CHB populations. (Supplementary Table S6 and Supplementary Figure S7). Many of these SNPs also show a clinal distribution in frequency wherein there is a flip of the ancestral allele from YRI to CHB and CEU populations (Table 1).

In parallel, we also analyzed iHS that measures the extended haplotype homozygosity of a given SNP along the ancestral allele relative to that of the derived allele^42^ in those 267 SNPs that have global F_ST_ > 0.3. We found that 33 SNPs (in 31 genes) had iHS > 2.0 in any of the three populations, indicative of positive selection (Fig. 4B), 14 of them being common to the 78 SNPs reported in Table 1. We observed that the DAF for most of these SNPs were higher in the European and Asian populations compared to the African, with a few of them almost reaching fixation (DAF > 0.9) (Table 1 and Supplementary Tables S6 and S7). We also checked whether the occurrence of 33 SNPs in 31 genes with global F_ST_ > 0.3 and iHS > 2.0 is non-random. Towards this, we created random sets of 30–35 genes (length normalized) and used their 3′UTR coordinates to count the total number of SNPs and SNPs with iHS > 2.0 in each of the three study populations. Our results highlight that the number of total SNPs and SNPs with iHS/global F_ST_ score is greater than mean + three times standard deviation in our test set of 31 genes when compared to the 1000 random sets of 30–35 genes (Supplementary Figure S8). This further substantiates that SNPs in exonized Alus are significantly enriched for signatures of selection (compared to random sets) and this is unlikely to be a chance event. We also observed that the Alu-miRNA targets in *RAD1* and *FKBP9* exhibited iHS > 2.0, and in total, we observed 63 such genes with the criteria of iHS > 2.0 as well as ΔDAF > 0.5 (Supplementary Table S7). These multiple evidences of signatures of positive selection in Alu-miRNA targets suggest that these sites might have assumed functional importance in a population-specific manner.

### Alu-miRNA targets create novel regulatory networks

#### Comparing Alu-miRNA sites in humans to other species

**Figure 5:**
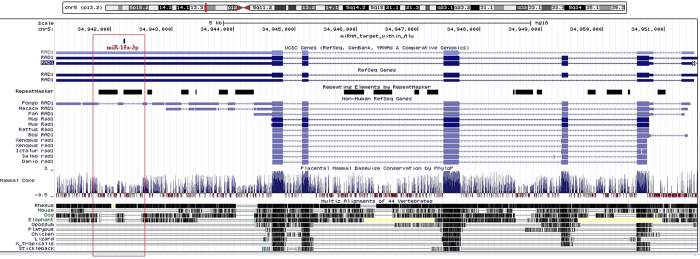
Gain of functional miRNA targets in humans through Alus. Lack of conservation for the Alu-miRNA targets was observed among human, chimpanzee, rhesus, gorilla, marmoset, orangutan, baboon and mouse for the majority of the functionally validated genes (Supplementary Table S8). Mouse (a non-primate) lacks Alu-miRNA target sites in the five genes studied here altogether. Additionally, chimpanzee and rhesus also do not have any of these Alu-miRNA targets. Gorilla, marmoset, orangutan and baboon have miRNA targets within repeat for any one of the gene. A representative figure from UCSC shows that all transcripts of *RAD1* in humans have an extended 3′UTR, harboring Alus with functional targets for miR-15a-3p. Other species have shorter 3′UTRs and they also show a low conservation at the Alu integration sites.

We next asked whether the presence of miRNA targets in primate-specific Alus provide novel regulatory motifs in existing networks. To explore this, we compared the conservation of our experimentally validated miRNA target sites among primates (human, chimpanzee, orangutan, gorilla, baboon, marmoset, rhesus) and mouse. We found that, in humans, the miRNA target sites for all the validated transcripts were present exclusively within the extended 3′UTR of Alu exonized isoforms (Supplementary Table S8). Rhesus and chimpanzee are devoid of miRNA targets within Alu for these genes. In the representative example of *RAD1*, we show how exaptation of Alu in the 3′UTR resulted in this novel functional Alu-miRNA target site (Fig. 5). There are a few primate species in which the genomic annotation is poor and hence, the status of Alu-miRNA target site cannot be conclusively determined. However, even for the organisms in which a BLAT search against the human gene sequence yielded a result, there were sequence level mismatches and patchy conservation. Alu-miRNA sites were either altogether absent or occurred in isolated cases (Supplementary Figure S9). Although miRNA target sites are present in a single gene for a few primates, it is only in humans that this entire novel regulatory network has evolved and is likely to be adaptive.

#### Functional impact of polymorphism within Alu-miRNA targets

We also explored the potential impact of the ‘high’ global F_ST_ SNPs on Alu-miRNA targets by using information from the PolymiRTS database. This evaluates whether a SNP has the potential to disrupt the conserved/non-conserved miRNA target sites by analyzing the change in TargetScan context scores^43^. A more negative context score difference indicates an increased likelihood that the target site is disrupted or a new target site is created by the derived allele. We could get information for 223 out of 267 ‘high’ global F_ST_ Alu-miRNA SNPs. We found that for 146 out of these 223 SNPs, the derived allele but not the ancestral allele, potentially changes (disrupts/ creates) the miRNA target site (Supplementary Table S9). We also investigated the possibility of these SNPs being present in a species-specific manner. From the data available in PolymiRTS, we found that 97 SNPs had both the alleles present only in human, while 40 SNPs had one human specific allele.

## Discussion

Alu elements, which are non-randomly distributed in the human genome, have been implicated in various regulatory functions at different cellular hierarchies^1^,^2^. In this study, we demonstrate that 3′UTR exonized Alus can participate in cellular response to stress through acquired miRNA binding sites in primates. Some of the targets are in alternate isoforms of the genes, suggesting these isoforms can be regulated differently due to presence/ absence of Alus. Also, it is possible that Alus in non-coding isoforms of genes functions to titrate miRNA levels in the cell by providing redundant miRNA binding sites^44^,^45^.

We validated miR-15a-3p targets in Alus and found that the target genes are involved in cell cycle and DNA damage response. We further show that the ectopic expression of this miRNA affects protein levels of these targets and modulate response to DNA damage and cell cycle progression in the cells. Our results indicate that although signatures of DNA damage begin to appear at 2.4 nM mimic treatment, yet its extent is not enough to elicit G1 arrest and hence, the cells easily cross the G1-S checkpoint. Once into the S phase, depletion of GTSE1 further accelerates their entry into mitosis and subsequent cell division. Overall the results indicate that an increased level of miR-15a-3p targets Alu-miRNA sites within *GTSE1* and *RAD1* to promote cell survival during stress response. The results of MTT, cell cycle progression and comet assays in no way claim that these are direct effects of RAD1 and/or GTSE1 downregulation. However, we believe that due to being hub genes involved in crucial cellular processes like genotoxic stress mitigation and cell division, their downregulation (which in this case is Alu-miRNA interaction mediated) can set rolling a complex cascade of cellular events culminating in DNA damage and cell proliferation.

miR-15a-3p is shown to be functionally involved in skin pigmentation^46^ and we have detected upregulation of this miR-15a-3p in skin cells. Interestingly, miR-15a-3p is also reported to be upregulated in response to UV exposure to skin and forms a regulatory network affecting extrinsic skin aging^47^. We believe that interaction of miR-15a-3p with Alus may have role in skin aging and adaptation of skin to stress in primates, similar to what has been shown before for interaction of exonized Alu with miR-661, in apoptosis^29^,^48^.

**Figure 6:**
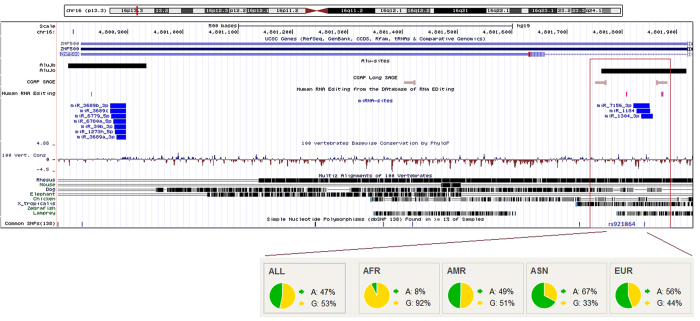
Representative scenario of Alu mediated regulatory events in a gene. Exonized AluJo present within the 3′UTR of *ZNF500* (a transcription factor) harbors A-to-I editing sites (human RNA editing database) as well as antisense expression of Alu (CGAP SAGE data). The same region also has three miRNA (miR-7156-3p, miR-1184 and miR-1304-3p) target sites, with a potential for cross-talk between these events. The presence of variation (SNP rs921864) adds an additional layer of regulation at the population level by disrupting the target site for miR-1184.

We observed that many such Alu-miRNA sites exhibit signatures of positive selection in the Phase-I 1000 Genomes data^34^. Since Alus have high frequency of A-to-I editing, the editing in functional Alu-miRNA sites can also affect miRNA-mediated regulation. One such example is the transcription factor *ZNF500*, where there is an evidence for A-to-I editing event in an AluJo family in the 3′UTR. The same site harbors binding sites for miR-7156-3p, miR-1184 and miR1304-3p and a SNP (rs921864) with pair-wise F_ST_ (YRI-CHB) 0.66, H < −20, iHS 2.46 in YRI and DAF > 0.5. The predicted functional consequence of this variation on the miRNA targetability (using miRanda) revealed that its presence in the binding site leads to the loss of miR-1184 target within Alu, while retaining the other two miRNA targets. We also observed that the derived allele frequency for this SNP is almost flipped in CEU and CHB compared to YRI, indicative of its functional adaptation in different populations (Fig. 6). The reverse is also true when the presence of SNP in the Alu-miRNA target leads to the gain of additional miRNA targets. For one of the functionally validated transcripts of *FKBP9*, we observe that a SNP (rs77844797) leads to the gain of targets for three additional miRNAs: miR-4647, miR-3689b-3p and miR-3689c.

Many genes showing evidence of selection in Alu-miRNA sites are involved in stress response or response to environmental challenges (Supplementary Table S7). Genes like *GFOD2* and *ANO8*, wherein the derived allele is nearing fixation in CEU and CHB, are known to be under selection pressure with *GFOD2* associated with diet induced changes in blood lipid of the Mexican population^49^,^50^. Similarly, mutations in *BCKDHB* gene (high DAF in CEU & CHB) are linked to cases of maple syrup urine disease, only in the Chinese and Korean populations^51^. Moreover, other genes related to DNA damage response/apoptosis (*GINS1, ICMT, TLK2, CRTAM, DNAJB7* and *TNFRSF9*), cell shape/integrity (*TBCCD1, BVES* and *GUCDI)* and neuro-protection (*NMNAT1*) also show signatures of positive selection for Alu-miRNA targets^52^.

Interestingly, many of the genes containing such highly differentiated SNPs in the Alu-miRNA sites within their 3′UTRs (listed in Table 1) have target sites (encompassing these SNPs) for miRNAs that are primate-specific (miR-661^29^, miR-1202^53^), human-specific (miR-4739, miR-5095)^54^, involved in the regulation of p53 signaling (miR-660^55^, miR-661^29^, miR-1285^56^) or in the apoptosis pathway (miR-17^44^,^45^, miR-30b^57^, miR-106a-3p^45^, miR-612^58^). This assumes importance from two different perspectives. Firstly, it highlights the fact that some of the miRNAs which have emerged in the primate lineage, have targets within primate-specific Alus and can regulate important physiological pathways. Secondly, multiple studies (including this, Fig. 2A) have demonstrated the functional importance of Alus in p53-mediated death pathways^29^,^59^. For instance, *FBXL20* contains a site for the human-specific miRNA miR-5095 in a recent AluY subfamily with a SNP whose frequency is completely flipped in CEU and CHB compared to YRI. This gene encodes an F-box containing E3 ubiquitin ligase which is involved in polyubiquitination and subsequent degradation of cyclins (D2 and D3); and its overexpression leads to mitotic arrest and induces apoptosis in leukemic cells^60^,^61^. Interestingly, 50% of the genes that contain SNPs exhibiting multiple signatures of positive selection (Table 1), along with our five experimentally validated genes (*RAD1, GTSE1, FKBP9, NR2C1* and *UBE2I*), were found to form a large interacting network, primarily connected through *UBC* (Supplementary Figure S10). This gene encodes a polyubiquitin precursor protein and just like p53, features multiple times in Alu-related events^12^. Protein degradation via ubiquitination forms an intrinsic component of cellular stress response and this happens in a rather coordinated fashion during apoptosis, along with cell cycle arrest, DNA damage sensing and invoking p53 signaling. It could very well be possible that exonized Alus provide “templates” upon which different kinds of regulations can act in a highly contextual manner to mediate this crucial crosstalk.

**Figure 7:**
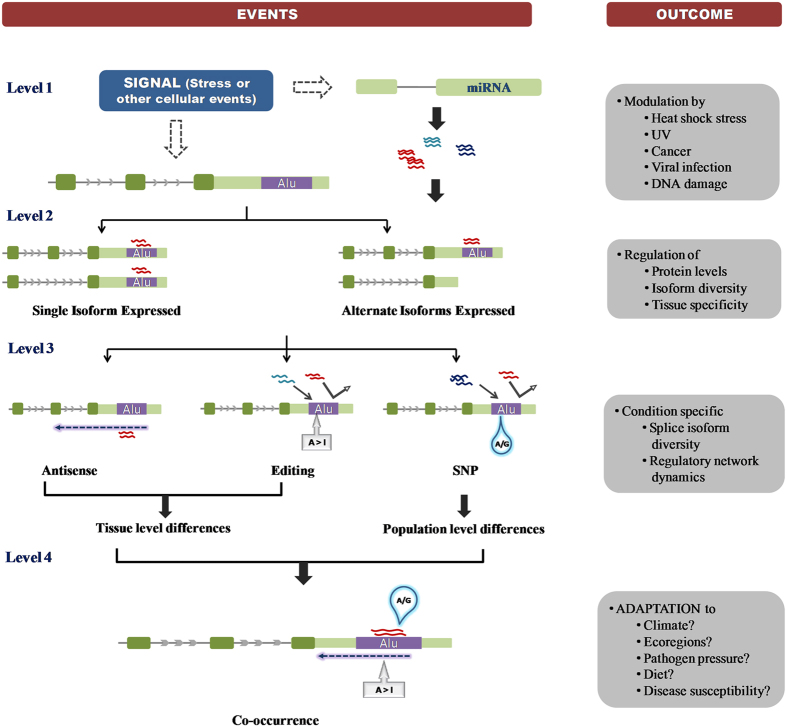
A model showing the functional outcomes of co-regulatory events occurring at exonized Alu. Stress and other cellular conditions can differentially modulate miRNA and transcript expression. miRNAs targeting Alu can regulate the global expression or isoform-specific expression of these Alu-exonized transcripts. Other Alu-mediated events, like antisense expression of Alu RNA or pre-mRNA A-to-I editing of Alu-miRNA sites, can further affect transcript expression and lead to tissue specificity through interaction with miRNAs. Presence of SNP at the Alu site can facilitate novel miRNA interaction outcomes at the population level. Furthermore, the cross-talk between all these events at the same locus can modulate or fine-tune the expression of gene in response to different environment conditions. Signatures of positive selection also suggest that variations in Alu-miRNA targets may also confer differences in adaptation and susceptibility to diseases.

Thus, the presence of functional Alu-miRNA targets within exonized transcripts increases their regulatory potential during stress response. This provides primate-specific mechanisms in maintaining cellular homeostasis (Fig. 7). Due to the potential of accumulating variations and A-to-I editing, functional Alu-miRNA sites can further expand the miRNA regulatory network. In future, it will be interesting to explore the functional consequences of *single Alu-multiple miRNAs* and *single miRNA-multiple Alu* targets, especially during different conditions of stress.

## Conclusion

Alus can act as transcriptional modulators^6^,^9^,^14^,^19^. When 3′UTR-resident exonized Alus harbor non-canonical miRNA target sites, they have the potential to add yet another mechanism of transcriptional modulation, in addition to exonization, A-to-I editing and antisense^12^. A large number of transcripts contain exonized Alus but it does not necessitate that all of them would respond to a particular cellular stimulus or be responsible for a specific phenotype. Rather it seems that the presence of Alu may confer plasticity to the expression level of the host genes^12^. Hence, selection for functional miRNA sites within Alu would not be operative at a global level i.e., may be present in one population but not in others. Rather, we propose, that it would act at the level of individual genes by providing a regulatory adapter that might be used depending on the function of the gene and how it responds to different cellular stimuli. The population level inferences are meant to emphasize how such gene-wise selection processes may have a role in modulating response to climatic adaptation/pathogen pressure/disease susceptibility/diet.

## Methods

A comprehensive flow diagram detailing all the steps (as well as the filtering criteria) used in the experimental work-flow of this paper has been provided as Supplementary Figure S1.

### Genome-wide expression profiling and qPCR

For both mRNA and miRNA genome-wide expression profiling, HeLa cells were used. Cells were obtained from the National Center for Cell Sciences, Pune, India and maintained in GlutaMax-DMEM high glucose medium supplemented with 10% heat-inactivated FBS, 25 mM HEPES and 1X antibiotic-antimycotic (GIBCO). Heat shock was induced by subjecting the cells to a temperature of 45 °C in a water bath for 30 mins, followed by recovery for two hours at 37°C in a humidified atmosphere containing 5%CO2 and 95% air. Total RNA, used for the expression profiling of both mRNA and miRNA, was isolated using Trizol (Invitrogen). Before microarrays, RNA was purified using RNeasy purification columns (Qiagen) and quantified (Agilent 2100 Bioanalyzer). RNAs with RNA Integrity Number (RIN) above seven were taken forward for the microarrays.

#### mRNA expression profiling

The data for mRNA whole genome expression profiling in response to heat shock, have been used from an earlier published work from our group^14^. Although details of the experiment are present in the published article, in summary, the mRNA profiling was done using Illumina Human Expression BeadChips (WG-6 v2.0). The arrays contain probes for ~47000 transcripts (~22000 annotated and rest being putative and predicted transcripts). Post analysis, an independent subset of differentially expressed genes from the microarray data was validated by qPCR with ~60% validation rate (primers listed in Supplementary Information).

#### miRNA expression profiling

Genome-wide expression profiling for miRNAs was done using dual-color miRCURY LNA miRNA Array (Exiqon). It consists of control probes, mismatch probes and 1769 capture probes, complementary to mature human, mouse, rat and their related viral sequences from v11.0 release of miRBase. Arrays also contain 435 proprietary human miRPlus sequences which are predicted miRNAs awaiting functional evidence. The capture probes are LNA enhanced oligonucleotides that result in high melting temperatures (Tm) of the probe-target duplex, thus enhancing the specificity and sensitivity of the array. Tm-normalized LNA™ probes bind to their target sequences with equal affinity regardless of the GC-content of the miRNA. This is further facilitated by varying the positions and amount of LNA™ in each probe. One μg of total RNA was used for 5′-dephosphorylation of miRNAs using calf intestinal phosphatase. Spike-in miRNAs were also added as an experimental control. Subsequently, samples were fluorescently labeled with Hy^3^ or Hy^5^ to the 3′-end of the miRNAs. The complementary samples (heat shock treated and untreated) labeled with Hy^3^ or Hy^5^ were combined on ice and added onto the slide placed in the slide chamber for hybridization. It was incubated for 16–18 hours in a water bath at 56 °C. Post-incubations, slides were washed rigorously and scanned immediately after drying using PerkinElmer scanner. All eight differentially expressed miRNAs and miRPlus, with targets within Alu-exonized transcripts, were validated by qPCR; of which two miRNA was validated (primers listed in Supplementary Information).

#### Quantitative Real-Time PCR (qPCR)

*mRNA* - One μg of total RNA was converted to cDNA using random primers from High Capacity cDNA Reverse Transcription kit (Applied Biosystems), as per manufacturer’s instructions. qPCR was performed using *KAPA SYBR^®^ FAST qPCR Kit* (KAPA Biosystems) on a 7900HT ABI platform. As the housekeeping gene, we used *RPL13A* for transcript specific qPCR. HPLC purified primers were synthesized from Sigma-Aldrich.

*miRNA* - cDNA synthesis was done using QuantiMir RT kit (Small RNA Quantitation system) from System Biosciences (SBI), following manufacturer’s protocol. Poly-A Tail was added to the small RNAs present in one μg of total RNA, followed by annealing of the anchor dT adaptor to the poly-A tail. These were then carried forward for cDNA synthesis resulting in a pool of anchor-tailed small RNAs. cDNA was checked by end-point PCR with universal reverse primer (kit supplied) and miRNA-specific forward primer. The cDNA was diluted 1:20 before being used for qPCR. As the reference small RNA, we found three small nucleolar RNAs - *SNORD38B*, *47* and *48* - to be invariant during heat shock. We have used *SNORD48* as the reference here.

All the experiments for miRNA as well as mRNA, both at the level of microarray and subsequent qPCR, were carried out in biological triplicates. The relative expression levels analyses for both mRNA and miRNA were carried out by the 2^−ΔΔ^CT method, using appropriate reference in each case.

### Functional validation of Alu-miRNA interaction

#### LNA modified Anti-miRs

For checking the target specificity of miR-15a-3p and miR-302d-3p, we designed LNA modified anti-miRs. The sequence of the oligos are, miR-15a-3p **-** 5′-**T**GAGG**C**AGCA**C**AATA**T**GGCC**T**G-3′ and miR-302d-3p **-** 5′-A**C**ACT**C**AAACA**T**GGAAG**C**ACT**T**A-3′. The nucleotides marked in bold are LNA modified. After transfection with the 50nM anti-miR (final concentration), qPCR was done for the target transcripts as well as these miRNAs. As an experimental control, we generated a 25-mer scrambled sequence, 5′-C**T**GCCGGAAG**T**CGA**T**TGC**C**CGA**C**GC-3′ which does not have any hit within the human genome. The anti-miRs as well as the scrambled oligo were synthesized from Exiqon.

#### Cloning of Alu-miRNA targets

Five targets showing rescue in the presence of anti-miR during heat shock response (*GTSE1, RAD1, NR2C1, FKBP9* and *UBE2I*) were cloned into the dual luciferase reporter construct, psiCHECK-2 (Promega, Madison, USA). psiCHECK2 vector contains Renilla luciferase as the primary reporter gene and firefly luciferase as the normalization control. We made two clones for each target transcript, only Alu (harboring the miRNA target site) and the complete 3′UTR. Templates were amplified from human genomic DNA followed by PCR purification (Qiagen), prior to cloning. The clones were confirmed by sequencing of template integration site, double digestion as well as PCR. Details of the primer sequences are provided in the Supplementary Information.

#### Transfections and reporter assays

Transient transfections were performed using Lipofectamine-2000 (Invitrogen) in OPTI-MEM medium (GIBCO). Twenty-four hours prior to transfection, 12-well plates were seeded with 2.5 × 10^5^ cells to achieve optimum confluency. 100 μM of anti-miR and control LNA were used for transfection. Transfection mix was removed five hours post-transfection and fresh media was added. Twenty-four hours after transfection, cells were subjected to heat shock stress. Following two hours incubation, RNA was isolated from treated and untreated cells for qPCR. Expression level of miRNA and target transcripts in heat shocked cells (no transfection) and harvested at the same time was used as reference to compare with anti-miR and control treatment conditions.

For transfecting the clones, transfection efficiency was optimized using pmaxGFP vector. Subsequently, 700 ng of each clone was used to transfect, in the presence and absence of 100 μM anti-miR. Empty vector was used as a control for all the experiments. Twenty-four hours after transfection, cells were lysed and luciferase activity was measured using dual luciferase assay (Promega). The luciferase activity was quantified using a Tecan Luminometer (Mannedorf, Switzerland).

For the overexpression experiments, mimics for miR-302d-3p and miR-15a-3p were synthesized by Sigma-Aldrich. The scramble oligo (negative control 1, HMC0002) was also synthesized by Sigma Aldrich based on miRBase ver20. The mimics as well as scrambled oligo used for ectopic overexpression experiments are not LNA modified. The lyophilized oligos were reconstituted in nuclease free water (Ambion). After serial dilution, 0.8 μM working dilutions were prepared and used in all subsequent transient transfections.

#### MTT assay

Nearly 10^4^ cells were seeded per well in a 96 well plate. Twenty-four hours post transfection, 100 μg of MTT (USB) was added to each well, followed by incubation at 37 °C in the dark for three hours. Subsequently the formazon crystals were dissolved in 100 μl DMSO (Sigma) and OD was measured at 540 nm (reference λ-620 nm) using Tecan luminometer. After subtracting the OD for blank wells, the viability of cells in the control (no-transfection) samples was considered to be 100% and transfected samples were normalized to it.

#### Cell cycle progression assay

Nearly 5 × 10^4^ cells were seeded per well in a 24-well plate. Twenty-four hours post transfection, cells were trypsinized and then resuspended in propidium iodide solution in PBS (two μg). Cells were incubated on ice for 15 mins, followed by centrifugation at 2000 rpm for 10 mins at 4 °C to pellet the cells. Using FACS (Accuri C6 Flow Cytometer), the cell population with high forward scatter and side scatter was gated and readings were taken using the FL2-A channel.

#### Comet assay

Twenty-four hours post transfection, nearly 3 × 10^4 ^ cells (in 1 X DPBS) were added to 0.75% low melting agarose. Single cell suspension was loaded onto glass slides (pre-coated with 0.1% low melting agarose) and allowed to cool at 4 °C. The cells were lysed by dipping slides into alkaline lysis buffer at 4 °C and washed in MilliQ. The slides were incubated in freshly prepared alkaline electrophoresis buffer at 4 °C and then run at 2 V/cm voltage and 300 mA for 20 mins. The excess alkali was neutralized with 0.4 M Tris and the slides were dried and 50 μM of propidium iodide was added to each slide just before imaging to stain the DNA. Images were obtained using a Leica fluorescence microscope at 40X. Both Olive Tail Moment and tail length were measured for 30 randomly selected comets per sample and analyzed using Komet 6.0 software (ANDOR technology, UK).

#### Western Blot

Twenty-four hours post-transfection, the cells were lysed using RIPA (Sigma-Aldrich), reconstituted with 100 mM Dithiothreitol and 1X Protease Inhibitor Cocktail (Sigma-Aldrich) in the ratio of 100:1:1. Subsequently the solution was centrifuged and the supernatant (total cell lysate, TCL) was collected and stored at −20 °C. Protein was estimated using Pierce BCA Protein assay kit (Fisher Scientific). 60 μg of TCL was loaded on the denaturing SDS-PAGE and was run at 80–100 V in Tris-Glycine Running Buffer. Proteins were transferred onto a 0.2 μm PVDF membrane (MDI) overnight at 30 V. The blot was checked with Ponceau S (USB) to confirm protein transfer and blocked using 1X NAP-Blocker (G-Bioscience) for four hours at room temperature, followed by overnight incubation in primary antibody (Supplementary Information) at 4 °C. Subsequently the blots were washed in 1X PBST, incubated in IgG-HRP conjugated secondary antibodies (Santa Cruz) for two hours at room temperature, again washed in 1X PBST and then developed in DAB (Biobasic). Images were analyzed with ImageJ software.

### Analysis of differentially expressed mRNAs and miRNAs

*mRNAs* - Illumina BeadChip expression arrays (WG-6 v2.0) were analyzed using Illumina BeadStudio with background subtraction and average normalization. For selection of the differentially expressed genes, we used the Illumina Custom error model and the recommended cutoff of ±13 differential score (corresponding to p-value 0.05). The details for this are mentioned in the earlier published work from our group^14^.

*miRNAs* - The *.gpr* files were obtained from Perkin Elmer scanner for Exiqon miRNA arrays. The respective dye-swapped samples were corrected for gene-specific dye-bias using the *GASSCO* method implemented in the “dyebias” BioConductor package^62^. For normalization, quantile and Loess methods were used. miRNA arrays have relatively few spots and unlike mRNA expression, miRNA expression levels can vary significantly between samples. Exiqon has used LNA-modified probes to have a small window of melting temperature to facilitate specific hybridization. But still, call rate for Exiqon array is less, around 40–45%. We got call rate of ~55% for our experiment. We analyzed the differentially expressed miRNAs based on the criteria of all those probes which have fluorescence value in minimum three arrays. Subsequently, we applied t-test and prioritized the miRNAs which have p-value less than 0.01 for experimental validation. We did not use imputation in this case as it carried far greater risk of false positives. The 32 differentially expressed miRNAs and miRPlus (p-value < 0.01) were further filtered for their targets within 3′UTR resident Alus in differentially expressed exonized transcripts (a subset of 4279 differentially expressed transcripts).

### miRNA targets within exonized Alus

#### Mapping Alu in differentially expressed genes

We retrieved the genic sequences for each of the differentially expressed transcripts using UCSC Table Browser (RefSeq genes track, hg18)^63^. In order to prevent genomic location ambiguity, we filtered out the sequences from the Table browser data that referred to chromosome type chr*_random and chr*_hap. The coordinates of Alu elements was obtained using the Repeat Masker track of Table Browser (hg18), which uses Repbase (a comprehensive database containing the consensus sequences of all annotated repeats) and UCSC (data and computational resources for the Pre-Masked Genomes)^64^.

#### miRNA target prediction

Target prediction for the differentially expressed miRNAs was done using two independent prediction tools, miRanda^65^ and TargetScan 5.1^66^. Potential targets were predicted by querying the 3′UTR database using following parameters for miRanda: gap open penalty of **−**8.0, gap extent of **−**2.0, score threshold of 50.0, energy threshold of **−**20.0 kcal/mol and scaling parameter of 4.0. miRanda searches for complementarity between miRNAs and their putative target using dynamic programming alignment and thermodynamic calculation. TargetScan (release 5.1) Perl script was downloaded and the potential 3′UTR targets were independently predicted. TargetScan makes use of evolutionary conservation, binding thermodynamics as well as other local sequence features (like accessibility) to predict miRNA targets, in addition to the minimal seed complement so as to improve the accuracy of its target prediction^66^. From the two separate lists of predicted targets generated by miRanda and TargetScan, we used a consensus of targets using custom Perl scripts. To reduce false positives, any 3′UTR that was predicted to be targeted by the same miRNA in both the prediction tools but at different positions, were excluded from the consensus list.

### Positive selection on Alu-miRNA targets

#### Whole genome prediction of miRNA targets in 3′UTR exonized Alu

As it was previously reported that the majority of Alu exonization happens in 3′UTR^12^, we downloaded the whole genome 3′UTR from UCSC genome browser (hg19) for all Alu-exonized and non-Alu exonized transcripts. For the genomic coordinates of 3′UTR exonized Alus in 7023 transcripts (2480 genes) out of total of 8480 (3177 genes), we used hg18 coordinates (as the earlier work was carried out using the same). The Alu sequences used in the analysis were further re-confirmed by RepeatMasker (standalone version) with >99.5% efficiency for being true Alu elements. miRanda (version 3.3) was used for prediction of target sites for 2578 miRNAs present in miRBase (release 20)^67^. Run parameters were made stringent by including *strict* 5-prime seed sequence match and score threshold of 100.0. miRNA hits within Alu were mapped back to their genomic coordinates. Subsequently, we calculated the miRNA targets that overlap with high F_ST_ SNPs across different categories such as miRNA target density within Alu and non-Alu region. We then performed Mann-Whitney test for data which was not normally distributed and calculated miRNA target density across different comparisons.

#### GeneMANIA based network analysis

We constructed a gene network (using human as the query species) for all the five genes in which Alu-miRNA target sites were functionally validated. GeneMANIA contains 2152 association networks from nine organisms for 166084 genes^35^. The network marks for physical interaction, co-expression, pathways, co-localization, genetic interaction, shared protein domains as well as prediction based interactions for a query gene set. The size of the nodes depicts their involvement in particular pathways and the edge width takes into consideration both the number of neighbors as well as type of interaction. Color of the edges indicate: Red – Physical interaction, Green - Genetic interaction, Light blue – Common pathway, Purple – Co-localization, and Grey – Final affected cellular pathways.

#### Signature of Positive selection using Phase-I 1000 Genomes data

The microarray experiments, its analysis and target prediction was done earlier with hg18 genome coordinates. Subsequently the Alu-miRNA target coordinates were converted into hg19 version using UCSC liftover tool^68^. For the analysis of selection, we used pre-calculated statistics on Phase-I 1000 Genomes dataset, present in 1000 Genomes selection browser database^34^. SNPs falling within Alu-miRNA coordinates were fetched from the data for all individuals available in 1000 Genomes selection browser for three populations – YRI (Yoruba in Ibadan, Nigeria), CEU (Utah Residents with Northern and Western Ancestry; CEPH) and CHB (Han Chinese in Bejing, China). To extract the SNPs falling in 3′UTR of Alu exonized genes, we overlapped the gene coordinates with SNPs in the 1000 Genomes Phase-I data^34^. The browser contains the reported SNPs and various statistical test results for selection and population differentiation. To investigate potential signature for positive selection, we focused on four frequency based approaches – global as well as pair-wise F_ST,_ Fay and Wu’s H, Tajima’s D and ΔDAF; and one long range haplotype based method - iHS. SNPs and corresponding population statistics were obtained for 3′UTR Alu-miRNA target genes. Two sets of analyses were performed in parallel and results for both are reported. The details of the selection analyses (along with the appropriate cut-offs applied at each step) have been summarized as a flowchart (Supplementary Figure S4). We used a step-wise filtering criteria: first a moderate global F_ST_ cutoff (>0.3), which was followed by either 1 or 2.

1. Pair-wise F_ST_ > 0.5 (between any population pair), followed by Fay and Wu’s H < −20 (in any of the three populations). For the 78 SNPs (in 60 genes) that pass these cut-offs, we had checked Tajima’s D values, iHS and global and population-wise ΔDAF scores as well. The miRNAs whose targets overlap these highly differentiated SNPs were also fetched (Table 1).
2. iHS (>2.0 in any of the three populations) and checked the ΔDAF distribution for 33 selected SNPs (in 31 genes) across all three study populations^40^.

We used the global F_ST_ values to identify the variants that show frequency differences among three populations: CEU (European), CHB (East Asian) and YRI (African). The global F_ST_ was calculated based on Weir and Cockerham’s F_ST_ estimator provided in 1000 Genomes selection browser database^34^. We have used a cut-off of global F_ST_ > 0.3, which we call as ‘high’ F_ST_ SNPs. Although an F_ST_ value greater than 0.65 is considered a measure of extreme population differentiation, we decided to go with this moderate cut-off since we have subsequently used a filtering approach to get to the Alu-miRNA regions with multiple signatures of selection (pair-wise F_ST_, Fay and Wu’s H, iHS, ΔDAF). Moreover, as expected, we did not find SNPs with F_ST_ > 0.65 within Alu-miRNA targets as these are non-canonical miRNA binding sites. Fay and Wu’s H statistics has been previously reported in a study dealing with positive evolution in miRNA binding sites^33^. Here we compared the H score of our region of interest (SNPs) to the adjoining flanking region of the genome. More negative this value of H, greater is the reduction in diversity in the positively selected regions^69^. We chose a cut-off value of less than −20 to infer positive selection signals. We have also reported here if some of the SNPs that have passed through multiple filters in the analysis, have a negative value for Tajima’s D. It indicates a deviation from neutrality which might be attributed to multiple factors, but when this data is viewed along with the H values for SNPs, it most likely indicates an event of recent positive selection. Also, since our analysis was specifically focused on Alu-miRNA target sites in which we expected moderate level of positive selection to be operative, we preferred using iHS in conjunction with F_ST_ over XP-EHH (XP-EHH is more useful when the selection operative on a population is nearing fixation whereas iHS has greater power to detect incomplete sweeps i.e., derived allele not yet fixed in the population^70^). ΔDAF denotes derived alleles that are at high frequency relative to other populations, indicative of the level of fixation of the derived allele. Thus, as additional criteria, we have checked for ΔDAF only in the final set of SNPs which had already qualified the criteria of global F_ST_ > 0.3 as well as pair-wise F_ST_ > 0.5, H < −20 or iHS > 2.0. Hence, it is likely that ΔDAF values, in this case, indicate possible positive selection^70^. The density of SNPs within the target sites were compared to that of the 3′UTR to infer selection constraint on the miRNA target sites within Alu. To calculate density, the length of the longest annotated UTR for each gene was taken into consideration. The target sites harboring signatures of selection were analyzed further for distribution within Alu subfamilies, miRNAs and gene categories.

### Modulation of Alu-miRNA regulatory network in humans

#### Cross-species comparison of the validated Alu-miRNA targets

For the five validated genes (*RAD1*, *GTSE1*, *NR2C1*, *FKBP9* and *UBE2I*), we downloaded the 3′UTR sequences of all the transcripts reported in UCSC for chimpanzee (panTro4), Orangutan (ponAbe2), Gorilla (gorGor3), Baboon (papAnu2), marmoset (calJac3), rhesus (rheMac3) and mouse (mm10)^47^. For the genes that lack any UCSC or RefSeq transcript, we have used the corresponding Ensemble transcript information. The target prediction was done using miRanda for the transcripts of all the seven species. The status of the target sites for the two validated miRNAs and their presence within or outside repetitive sequences was analyzed.

#### Analyzing functional impact of SNPs on Alu-miRNA target sites

PolymiRTS (Polymorphism in microRNAs and their TargetSites, version 3.0) was used to evaluate the functional impact of the variations within Alu-miRNA targets^43^. This database catalogues DNA level polymorphisms in miRNA seed regions as well as their target sites. We have run it on the default parameters. For a query SNP (rsID), the database returns the number of miRNAs that overlap with the rsID, the number of species in which the target is conserved and the number of miRNA sites disrupted by the presence of that SNP allele (Supplementary Table S9). PolymiRTS uses TargetScan defined ‘context score’ (calculated using a combination of the above three parameters) for inferring effect of variation on miRNA target. In essence, for the total number of miRNA targets spanning a particular SNP, PolymiRTS returns the number of miRNAs for which the context score is negative. The difference in the context score was used to infer whether the SNPs disrupt the miRNA target sites^60^. More negative context score indicates an increased likelihood that the target site is disrupted or a new target site is created by the derived allele. The information about ancestral allele was also obtained.

### Online Data Resource

The datasets supporting the results of this article are available in the Gene Expression Omnibus (GEO) repository. Illumina mRNA BeadChip data is available at GSE26776 and Exiqon miRNA expression data is available from GSE60472.

## Additional Information

**How to cite this article**: Pandey, R. *et al*. Alu-miRNA interactions modulate transcript isoform diversity in stress response and reveal signatures of positive selection. *Sci. Rep.* **6**, 32348; doi: 10.1038/srep32348 (2016).

## Supplementary Material

Supplementary Information

Supplementary table S1

Supplementary table S2

Supplementary table S3

Supplementary table S4

Supplementary table S5

Supplementary table S6

Supplementary table S7

Supplementary table S8

Supplementary table S9

## Figures and Tables

**Table 1:** 78 SNPs (in 60 genes) within Alu-miRNA target sites exhibit multiple signatures of positive selection.

Gene	SNP	Pair-wise F_ST_			Fay_Wu’s H			Tajima’s D			DAF		
		YRI_CHB	YRI_CEU	CEU_CHB	CEU	CHB	YRI	CEU	CHB	YRI	CEU	CHB	YRI
*AARS2*	rs325011	**0.515**	0.431	0.033	**−22.591**	2.921	4.865	**−0.570**	1.367	−**0.256**	0.965		0.494
*ANAPC16*	rs6480601	**0.598**	0.161	0.286	**−22.942**	−10.310	1.234	**−1.396**	**−0.441**	0.192	0.300	**0.711**	0.068
*BCKDHB*	rs1811844	**0.576**	**0.671**	0.012	**−38.938**	−19.428	7.135	1.177	2.415	1.659	**0.824**	0.747	0.114
*BVES*	rs221655	**0.547**	**0.604**	0.002	**−35.485**	**−31.006**	12.059	**−1.424**	**−1.052**	**−0.038**	0.112	0.155	**0.767**
*CD209*	rs12460694	0.356	**0.520**	0.352	**−36.930**	−11.461	−19.954	0.197	1.798	0.143		0.371	
*CEP104*	rs7528951	**0.868**	0.395	0.380	−4.847	−5.501	**−23.971**	1.174	1.139	**−0.288**	0.453	0.041	**0.915**
*CMBL*	rs10076725	0.229	0.194	**0.594**	−19.559	**−56.996**	−16.407	0.535	**−1.263**	0.747	**0.700**	0.072	0.369
*DDX51*	rs11246938	**0.594**	**0.518**	0.006	**−20.098**	−10.953	−0.296	**−1.375**	**−0.993**	**−0.631**	0.124	0.077	**0.710**
*DDX51*	rs12424892	**0.584**	**0.533**	0.001	**−20.098**	−10.953	−0.296	**−1.375**	**−0.993**	**−0.631**	**0.900**	**0.928**	0.307
*DHRS4-AS1*	rs112851730	**0.597**	0.469	0.055	**−75.557**	**−79.678**	**−22.540**	**−1.110**	**−2.340**	1.297	**0.929**	**0.995**	0.403
*DHRS4-AS1*	rs113551151	**0.603**	0.476	0.055	**−75.557**	**−79.678**	**−22.540**	**−1.110**	**−2.340**	1.297	0.071	0.005	**0.602**
*DPPA4*	rs3792321	**0.657**	0.431	0.100	−7.961	−2.900	**−32.118**	1.955	1.349	0.436	0.465	**0.696**	0.017
*DUSP19*	rs10200193	**0.705**	0.206	0.342	1.123	**−33.184**	8.978	0.422	**−1.361**	**−0.419**	**0.535**	**0.933**	0.205
*EEF1E1-BLOC1S5*	rs2748375	**0.658**	0.260	0.267	−10.041	**−52.521**	−3.838	2.184	0.019	0.793	0.300	**0.696**	0.017
*EGF*	rs7653900	0.031	**0.610**	0.460	**−27.671**	4.260	−2.875	**−1.107**	1.438	**−0.173**	**0.912**	0.381	0.256
*FBXL19-AS1*	rs12930657	**0.781**	0.426	0.237	−2.038	**−24.287**	0.098	0.982	**−2.090**	**−0.608**	**0.700**	**0.969**	0.182
*FBXL20*	rs4325601	**0.523**	0.455	0.003	−15.787	**−20.654**	9.685	1.005	**−0.167**	**−0.544**	**0.718**	**0.773**	0.176
*FOXL2NB*	rs7649365	0.479	**0.678**	0.103	**−46.982**	**−33.494**	4.819	**−2.276**	**−1.161**	0.100	**0.982**	**0.845**	0.290
*FTLP10*	rs11131742	**0.562**	0.162	0.261	−9.842	**−43.181**	−14.138	1.605	**−2.103**	0.229	0.265	0.005	**0.563**
*GINS1*	rs6037121	**0.538**	0.013	0.436	6.849	**−44.913**	9.490	1.482	**−0.883**	**−0.293**	0.459	**0.938**	0.364
*GPSM2*	rs12743716	**0.597**	0.240	0.206	−3.468	**−34.839**	9.503	0.276	**−1.361**	0.096	**0.729**	**0.969**	0.358
*HES2*	rs4908889	**0.683**	**0.524**	0.054	**−38.218**	**−24.711**	1.574	**−1.022**	**−0.268**	0.368	**0.835**	**0.943**	0.239
*HSH2D*	rs432781	**0.594**	0.230	0.205	−14.433	**−28.230**	**−22.427**	0.662	**−1.066**	**−0.251**	**0.700**	**0.954**	0.335
*HTATSF1P2*	rs4269412	0.357	**0.661**	0.149	**−63.325**	−17.117	**−34.270**	0.292	2.050	0.862	**0.659**	0.371	
*ITPRIPL2*	rs57236534	0.406	**0.571**	0.063	**−30.751**	−9.522	0.460	**−1.788**	**−0.660**	0.033	**0.965**	**0.856**	0.364
*IVD*	rs2075625	**0.599**	0.193	0.232	8.014	**−22.145**	−8.386	1.617	**−0.148**	**−0.562**	**0.541**	0.191	**0.847**
*IVD*	rs4923865	**0.597**	0.315	0.119	8.014	**−20.713**	−5.243	1.617	**−0.113**	**−0.434**	**0.600**	**0.830**	0.176
*IVD*	rs11630878	**0.605**	0.194	0.239	9.854	**−20.713**	−5.243	1.613	**−0.113**	**−0.434**	0.459	**0.814**	0.153
*IVD*	rs11630850	**0.605**	0.194	0.239	9.854	**−20.713**	−5.243	1.613	**−0.113**	**−0.434**	0.459	**0.814**	0.153
*LILRA6*/ *LILRB2*/ *LILRB3*	rs2361804	**0.612**	0.313	0.142	−12.116	**−51.447**	−11.251	2.144	0.532	1.716	0.300	0.082	**0.733**
*LOC105447645*	rs570794	**0.566**	0.003	**0.531**	−7.253	**−41.168**	−9.092	1.619	**−1.065**	1.351	0.453	**0.985**	0.415
*LOC105447645*	rs571689	**0.566**	0.003	**0.531**	−7.253	**−41.168**	−9.092	1.619	**−1.065**	1.351	0.453	**0.985**	0.415
*LOC105447645*	rs507711	**0.502**	0.004	**0.531**	−5.990	**−41.168**	−9.092	2.050	**−1.065**	1.351	**0.547**	0.015	**0.523**
*MRPS10*	rs3199639	**0.539**	0.388	0.033	−5.530	**−33.620**	−14.099	1.878	0.015	**−0.469**	0.359	0.232	**0.841**
*MRPS10*	rs9381159	**0.572**	0.388	0.051	−5.530	**−33.620**	−14.099	1.878	0.015	**−0.469**	0.359	0.206	**0.841**
*MS4A10*	rs12577187	**0.519**	0.004	**0.539**	5.805	**−21.114**	1.036	0.666	**−0.562**	**−0.736**	0.071	**0.665**	0.085
*NMT2*	rs2400118	**0.723**	**0.557**	0.055	**−34.558**	**−36.623**	5.111	**−1.017**	**−0.974**	**−0.098**	**0.706**	**0.851**	0.097
*NOL9*	rs10158065	0.337	**0.531**	0.060	**−62.005**	**−42.239**	−2.763	**−0.661**	0.708	0.670	**0.894**	**0.753**	0.301
*NOL9*	rs11122049	0.337	**0.529**	0.058	**−62.005**	**−42.239**	−2.763	**−0.661**	0.708	0.670	**0.888**	**0.747**	0.296
*PSMB2*	rs12082263	0.002	**0.721**	**0.666**	**−41.524**	**−34.361**	−16.777	**−2.653**	**−1.647**	**−1.155**	**0.894**	0.186	0.142
*PSMB2*	rs11264180	0.478	0.077	**0.647**	**−41.524**	**−34.361**	−16.777	**−2.653**	**−1.647**	**−1.155**	0.029	**0.706**	0.148
*RAB3B*	rs4537499	**0.582**	0.304	0.115	**−22.665**	**−27.025**	−3.451	**−1.002**	**−1.200**	**−0.501**	**0.618**	**0.840**	0.199
*RNF168*	rs9872866	**0.677**	0.157	0.367	−2.861	**−20.418**	12.103	0.419	**−0.451**	**−0.852**	0.359	**0.825**	0.108
*RNF168*	rs59487085	**0.609**	0.085	0.372	−2.861	**−20.418**	12.103	0.419	**−0.451**	**−0.852**	0.459	**0.902**	0.250
*RNF207*	rs11121500	0.492	**0.549**	0.005	**−38.594**	**−31.318**	5.294	**−1.766**	**−1.173**	0.431	**0.924**	**0.881**	0.324
*RPP14*	rs3210776	**0.791**	0.272	0.422	−12.302	**−51.579**	−9.828	1.159	**−0.921**	0.137	0.300	**0.814**	0.011
*RPP14*	rs1135089	**0.806**	0.299	0.422	−11.166	**−45.431**	−12.550	1.339	**−0.485**	0.216	0.300	**0.814**	
*RSU1*	rs45461296	0.351	**0.545**	0.060	**−24.623**	−15.202	−6.458	**−1.627**	**−1.036**	**−0.671**	**0.894**	**0.753**	0.290
*RSU1*	rs45514294	0.372	**0.538**	0.041	**−24.623**	−15.202	−6.458	**−1.627**	**−1.036**	**−0.671**	**0.871**	**0.747**	0.267
*S1PR3*	rs1129925	**0.641**	0.219	0.253	−17.269	**−27.133**	−6.914	**−0.631**	**−0.653**	**−1.032**	0.453	**0.820**	0.131
*SEPN1*	rs3203750	**0.524**	0.205	0.165	−11.257	**−48.385**	3.951	0.973	**−1.548**	0.086	**0.747**	**0.959**	0.409
*SLC28A2*	rs2458225	**0.681**	0.017	**0.578**	−2.327	**−30.633**	5.342	2.017	**−1.036**	0.435	0.329	**0.938**	0.233
*SLC35E2*	rs61777506	0.304	**0.603**	0.140	**−33.670**	−7.388	7.536	0.473	1.735	0.105	0.071	0.289	**0.716**
*SMIM12*	rs11263953	**0.677**	0.035	**0.535**	−2.822	**−27.384**	−0.592	1.154	**−0.946**	0.126	0.377	**0.943**	0.244
*SMIM12*	rs6697614	**0.653**	0.433	0.106	−2.822	**−27.384**	−0.592	1.154	**−0.946**	0.126	**0.835**	**0.974**	0.313
*STAT2*	rs4996382	**0.719**	**0.669**	0.020	**−23.481**	−7.815	−11.120	**−2.178**	**−1.995**	**−0.093**	0.024		**0.710**
*TMEM40*	rs4684873	**0.621**	0.212	0.269	0.313	**−27.455**	−5.997	0.874	**−0.510**	**−1.120**	0.288	0.686	0.034
*TMPRSS11B*	rs13125514	**0.625**	0.228	0.261	−13.452	**−43.633**	−10.160	1.170	**−2.222**	**−0.604**	**0.735**	0.995	0.375
*TNFRSF25*	rs3007418	**0.651**	**0.548**	0.050	−14.520	**−20.331**	0.138	**−1.266**	**−1.810**	**−0.696**	**0.053**		**0.642**
*TNFRSF9*	rs12564367	0.228	0.196	**0.556**	**−22.980**	−1.339	−13.523	**−0.593**	1.016	**−0.610**	0.982	0.397	**0.756**
*TP73-AS1*	rs1181864	0.036	**0.711**	**0.582**	**−26.005**	−9.417	−3.538	**−0.347**	0.676	**−0.250**	0.188	**0.830**	**0.926**
*TRIP13*	rs6555582	0.288	**0.712**	0.262	**−33.963**	−0.155	−0.175	**−2.277**	**−0.839**	0.056			
*TSPAN16*	rs411834	0.396	**0.501**	0.022	**−26.646**	−7.999	3.643	**−0.730**	0.365	0.324	0.059	0.129	**0.608**
*TSPAN16*	rs445026	0.403	**0.507**	0.022	**−26.646**	−7.999	3.643	**−0.730**	0.365	0.324	**0.941**	**0.871**	0.386
*TSPAN16*	rs445169	0.396	**0.501**	0.022	**−26.646**	−7.999	3.643	**−0.730**	**0.365**	0.324	**0.941**	**0.871**	0.392
*VHL*	rs1136249	**0.571**	0.442	0.024	−11.630	**−28.103**	5.172	0.595	**−0.693**	**−0.382**	**0.694**	**0.799**	0.165
*VHL*	rs1681668	**0.513**	0.387	0.021	−11.630	**−28.103**	5.172	0.595	−0.693	**−0.382**	**0.694**	**0.794**	0.205
*VSTM4*	rs4240499	**0.755**	**0.527**	0.105	−16.215	**−22.284**	12.465	0.030	0.400	0.094	**0.624**	**0.835**	0.057
*ZDHHC8P1*	rs12485210	**0.506**	0.357	0.035	**−80.877**	**−69.594**	**−42.037**	**−0.058**	0.275	0.247			
*ZDHHC8P1*	rs6003681	**0.506**	0.357	0.035	**−80.877**	**−69.594**	**−42.037**	**−0.058**	**0.275**	0.247	0.171	0.077	**0.631**
*ZNF106*	rs1139100	**0.840**	**0.863**	0.005	−9.112	**−24.650**	4.460	**−0.488**	**−0.853**	**−0.317**	**0.877**	**0.861**	0.011
*ZNF175*	rs4801879	**0.663**	0.096	**0.507**	2.295	**−54.041**	−5.147	1.530	**−0.264**	0.377	0.100	**0.675**	
*ZNF197*	rs9865162	**0.555**	0.079	0.323	−4.990	**−20.126**	−15.882	**−0.637**	−1.949	**−1.178**	0.424	**0.845**	0.227
*ZNF500*	rs921864	**0.660**	0.457	0.076	−11.268	**−20.000**	1.246	1.070	1.503	0.094	**0.541**	**0.737**	0.045
*ZNF528*	rs8106133	0.181	**0.725**	0.391	**−50.846**	−18.850	−4.816	**−1.064**	0.594	0.263	**0.818**	0.325	0.068
*ZNF670*	rs12144260	**0.510**	0.031	0.377	4.999	**−40.482**	12.912	0.966	**−1.833**	0.227	0.377	0.005	**0.511**
*ZNF696*	rs7387702	**0.688**	0.400	0.158	2.706	**−25.135**	−16.796	2.837	2.149	**−0.008**	0.424	**0.717**	0.011
*ZNF860*	rs13094125	0.419	**0.543**	0.018	**−38.882**	−1.324	**−37.144**	**−0.121**	2.272	**−0.886**	**0.788**	**0.691**	0.176

All of them have qualified global F_ST_ > 0.3, pair-wise F_ST_ > 0.5 (between any population pair; highlighted in bold) and Fay and Wu’s H < −20 (in any of the three populations; highlighted in bold). Additionally, 70 SNPs have a negative value for Tajima’s D (highlighted in bold) and many of them have DAF > 0.5 (highlighted in bold) in any of the three study populations. The data in the table have been taken from 1000 Genome Phase-I^34^. The miRNAs whose targets overlap with these 78 SNPs, the Alu subfamilies in which these Alu-miRNA target sites occur, the information for the ancestral and derived alleles and the global minor allele frequency (MAF; fetched from dbSNP which uses 1000 Genomes Phase-3 data) have been provided in Supplementary Table S6.
