# Supplementary material for: Alu-miRNA interactions modulate transcript isoform diversity in stress response and reveal signatures of positive selection
Source: Sci Rep. 2016 Sep 2;6:32348. doi: 10.1038/srep32348 (PMC5009348; doi:10.1038/srep32348)
Supplement: Supplementary Information [file srep32348-s1.pdf]

Alu-miRNA interactions modulate transcript isoform diversity in stress response and reveal signatures of positive selection

Rajesh Pandey[1], Aniket Bhattacharya[2,3#], Vivek Bhardwaj[2#], Vineet Jha[4#], Amit K. Mandal[5], Mitali Mukerji[1,2,3,5*]

[#] Equal contribution

[*]Corresponding author

Primers used for validation of Illumina microarray data		
**S. No.**	**Genes**	**Primer sequence**
1	B2M_FP	**5'-** TGCTGTCTCCATGTTTGATGTATCT **-3'**
2	B2M_RP	**5'-** TCTCTGCTCCCCACCTCTAAGT **-3'**
3	CREG1_FP	**5'-** CAGAAGTTGGTCTGTGCCAA **-3'**
4	CREG1_RP	**5'-** GCGTGCCCTATTTCTACCTG **-3'**
5	IGFBP2_FP	**5'-** GAGCAGGTTGCAGACAATGG **-3'**
6	IGFBP2_RP	**5'-** CGGCCAGCTCCTTCATAC **-3'**
7	ANAPC1_FP	**5'-** ATGGTGCCTAGTTTTGCAGC **-3'**
8	ANAPC1_RP	**5'-** GCTGGCGACTCTCAATATCC **-3'**
9	CTPS2_FP	**5'-** CCCAGTCTCATTGTTCCTCC **-3'**
10	CTPS2_RP	**5'-** CCACAGAGTTTAGGCCAAATG **-3'**
11	ILF3_FP	**5'-** AGGCCTACGCTGCTCTTGCT **-3'**
12	ILF3_RP	**5'-** GCCGAAGCCAGGGTTATGTG **-3'**
13	LAMC3_FP	**5'-** CGCTTGTAGATGGCAAAGC **-3'**
14	LAMC3_RP	**5'-** CCACCTCGGTCAACATCAC **-3'**
15	SPTAN1_FP	**5'-** CTGCTGTTTCCAGCACTTTG **-3'**
16	SPTAN1_RP	**5'-** GAGGCTCCTCGGTCCTTC **-3'**
17	WDR62_FP	**5'-** GAAAGGATCCTGATGGCAAA **-3'**
18	WDR62_RP	**5'-** GGTCAGAGCTCTTCCACAGC **-3'**
19	RPL13A_FP	**5'-** GTTGATGCCTTCACAGCGTA **-3'**
20	RPL13A_RP	**5'-** AGATGGCGGAGGTGCAG **-3'**

Supplementary S1. Primers used for SYBR-based qPCR validation of the differentially expressed transcripts from Illumina microarray data. *RPL13A* was used as the reference gene.

Primers for validation of Exiqon microarray data		
**S. No.**	**miRNAs**	**Primer sequence**
1	miR-302d-3p_FP	**5'-** ACACTCAGCTGGTAAGTGCTTCCATGTTT **-3'**
2	miR-15a-3p_FP	**5'-** ACACTCAGCTGTTAGCAGCACATAATGG **-3'**

Supplementary S2. Primers used for SYBR-based qPCR validation of differentially expressed miRNAs: miR-302d-3p and miR-15a-3p.

Primers for reference small RNA		
**S. No.**	**Reference small RNAs**	**Primer sequence**
1	SNORD38B_FP	**5'-** AAAGTGTGTCTGAGGAGA **-3'**
2	SNORD47_FP	**5'-** CCGTTCCATTTTGATTCTGAG **-3'**
3	SNORD48_FP	**5'-** TAACTCTGAGTGTGTCGCTGA **-3'**

Supplementary S3. Primers for *SNORD* RNAs (invariant between heat shock treated and untreated condition). Out of these, *SNORD48* was used as the reference for SYBR-based qPCR validation of miRNAs.

Primers for validation of Alu-miRNA target transcripts		
**S. No.**	**Target genes**	**Primer sequence**
1	ADD1_FP	**5'-** CAAAGCATGCTCAGAAATGG **-3'**
2	ADD1_RP	**5'-** CTGGGATGACAGGCATCAG **-3'**
3	UBE2I_FP	**5'-** TGCAGATGCGAGTCTGTTTC **-3'**
4	UBE2I_RP	**5'-** CTGGATCTCACAGCCTCTCC **-3'**
5	RAD1_FP	**5'-** CACCACTCCTCCTTCTTCCA **-3'**
6	RAD1_RP	**5'-** AATGCAAAGTGTGTGCAAGC **-3'**
7	GTSE1_FP	**5'-** GAGTCATGAAGCCAGAGAAGC **-3'**
8	GTSE1_RP	**5'-** GCCAGGATGGTCTTGATCTC **-3'**
9	FHL2_FP	**5'-** ACGAAGCAGGGACATACAGG **-3'**
10	FHL2_RP	**5'-** GGGGATACCCACCATTTTCT **-3'**
11	FKBP9_FP	**5'-** ACCACACCCTGCACAGAACT **-3'**
12	FKBP9_RP	**5'-** AATGGGCAGAGAAACAAGGA **-3'**
13	NR2C1_FP	**5'-** GCCAGAACACAAGACACCAA **-3'**
14	NR2C1_RP	**5'-** CTGCCTGCCCAGTCACTT **-3'**

Supplementary S4. Primers used for qPCR validation of Alu-miRNA targets within Alu-exonized transcript isoforms.

hsa-miR-15a-3p: **5'- TGAGGCAGCACAATATGGCCTG -3'**

hsa-miR-302d-3p: **5'- ACACTCAAACATGGAAGCACTTA -3'**

LNA-control: **5'- CTGCCGGAAGTCGATTGCCCGACGC -3'**

- All the nucleotides in Red are LNA modified for increased stability.

Supplementary S5. LNA-modified Anti-miRs for miR-15a-3p and miR-302d-3p, along with control (scrambled) oligo sequence.

**Primers for cloning of Alu-miRNA targets**		
**S. No.**	**Genes**	**Primer sequence**
1	UBE2I_Clone_Alu_FP	5'- GAAGTCACAACGGAAGAGGTG -3'
2	UBE2I_Clone_Alu_RP	5'- TCCTTTACCAGGTCCTGTGC -3'
3	UBE2I_Clone_3'UTR_FP	5'- ATTATCCATCTTCGCCACCA -3'
4	UBE2I_Clone_3'UTR_RP	5'- TCCTTTACCAGGTCCTGTGC -3'
5	NR2C1_Clone_Alu_FP	5'- GCAGAAAATTGTTTTTGAGAATAAGC -3'
6	NR2C1_Clone_Alu_RP	5'- TGAAACATTTTGGGCTCAATTA -3'
7	NR2C1_Clone_3'UTR_FP	5'- TGAAAATGGAGCCTGCAGAT -3'
8	NR2C1_Clone_3'UTR_RP	5'- GCACAATGGAACAAACGAAG -3'
9	FKBP9_Clone_Alu_FP	5'- CACCTGCCTTCCTCACTAGC -3'
10	FKBP9_Clone_Alu_RP	5'- GCTTCAATATTTTGGCTCTCAAG -3'
11	FKBP9_Clone_3'UTR_FP	5'- TCCACATTGCTTGAAACAGG -3'
12	FKBP9_Clone_3'UTR_RP	5'- ACGTGCTTTTGACTTTGGTG -3'
13	GTSE1_Clone_Alu_FP	5'- TTTCAACCCTCAGAAACAAGC -3'

14	GTSE1_Clone_Alu_RP	5'- CCTCTCCAGCACTGGGAATA -3'
15	GTSE1_Clone_3'UTR_FP	5'- CACCTCCTCCACTCTGCTCT
16	GTSE1_Clone_3'UTR_RP	5'- GCAGGACTGGCTGAAAGTCT -3'
17	RAD1_Clone_Alu_FP	5'- CCAATTTCAGATTTTCTTTTAGCA -3'
18	RAD1_Clone_Alu_RP	5'- TCCTTACTGAGCTGATTTATATTTGTT -3'
19	RAD1_Clone_3'UTR_FP	5'- TGCCCTGATGAAGAAGTTCC -3'
20	RAD1_Clone_3'UTR_RP	5'- AAAGCAGCACTGCCTATTCC -3'

Supplementary S6. List of primers used for cloning of Alu-miRNA targets. The Alus harboring the miRNA target as well as the corresponding complete 3'UTRs were cloned.

**Company**	**Catalog No.**	**Description**	**Dilution used**
Abcam	ab5363	Mouse monoclonal to Rad1 [4126]	1:1000
Abcam	ab103232	Rabbit polyclonal to GTSE1	1:500
Sigma	T6199	Monoclonal Anti-α-Tubulin antibody produced in mouse	1:2000
Santa Cruz	sc-2004	Goat anti-rabbit IgG-HRP	1:2000
Santa Cruz	sc-2005	Goat anti-mouse IgG-HRP	1:2000

Supplementary S7. Details of the antibodies used for Western Blot

(A)

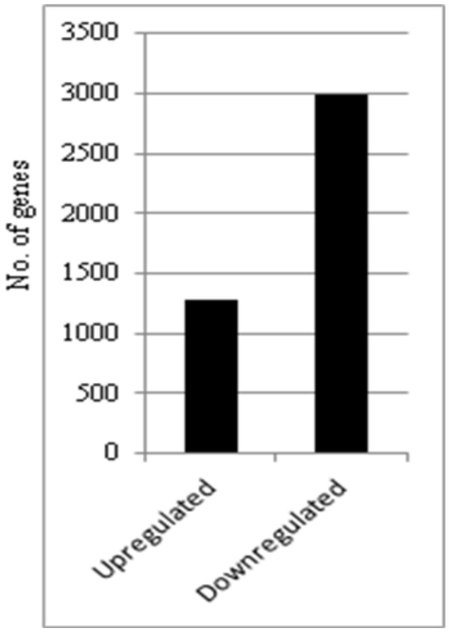

(B)

miRNA	Expression
hsa-miR-302d-3p	Upregulated
hsa-miR-15a-3p	Upregulated
hsa-miR-526b*	Upregulated
hsa-miR-1264	Upregulated
hsa-miR-27b	Downregulated
hsa-miR-601	Downregulated
hsa-miRPlus-D1120	Upregulated
hsa-miRPlus-F1022	Upregulated

Supplementary S8. Total number of differentially expressed mRNAs and miRNAs in response to heat shock stress following genome-wide expression profiling using HeLa cells. **(A)** Differentially expressed mRNAs; **(B)** Differentially expressed miRNAs.

Supplementary Table Legends

Supplementary table S1: List of 4279 differentially expressed transcripts (and corresponding genes) and 32 differentially expressed miRNAs (22 annotated miRNAs and 10 miRPlus; Exiqon microarray data) in response to heat shock stress (analyses have been done using genome version hg18).

Supplementary table S2: Genome-wide targets for miR-302d-3p and miR-15a-3p in the 3'UTR of transcripts (analyses have been done using genome version hg18).

Supplementary table S3: Targets for miR-302d-3p and miR-15a-3p in the downregulated genes. Also contains information for targets present exclusively within Alu (analyses have been done using genome version hg18).

Supplementary table S4: Dual luciferase (DLR) assay data for the Alu-miRNA target clones for miR-15a-3p targets (*RAD1, NR2C1, FKBP9* and *GTSE1*) and miR-302d-3p target (*UBE2I*).

Supplementary table S5: Comparative analyses of the occurrence of high global F_{ST}/ high iHS SNPs in the Alu vs. the non-Alu regions of the 3'UTR.

Supplementary table S6: It has information for genome-wide miRNA targets in exonized Alus (derived from miRanda) and corresponding SNPs (from 1000 Genomes selection browser). Different lists have: 1) Alu_miRNA_SNP: miRNA target genes and Alus with SNPs, 2) Pop_stats_High global F_{ST}: Population-wise F_{ST}, iHS and ∆DAF for three 1000 genome populations for the SNPs with high global F_{ST} values (>0.3), 3) iHS_selected: Population-wise details for SNPs selected on the basis of iHS (>2.0), 4) ∆DAF_of_selected: Analysis of population-wise DAF of selected SNPs, 5) Alu-miRNA Stats: Genes with more than one miRNA target and miRNAs targeting more than one gene, 6) Pairwise F_{ST}>0.5: 178 SNPs (selected on the basis of pair-wise F_{ST}>0.5 between any two populations among YRI, CEU & CHB) with their population statistics – global and pair-wise F_{ST}, population-wise iHS, global and population-wise ∆DAF, Tajima's D and Fay-Wu's H scores, 7) Pairwise F_{ST}>0.5, iHS>2.0: 29 SNPs (in 21 genes) that remain after applying the filter of pair-wise F_{ST}>0.5 as well as iHS>2.0 and their population statistics – global and pair-wise F_{ST}, population-wise iHS, global and population-wise ∆DAF, Tajima's D and Fay-Wu's H scores for CEU, CHB and YRI populations, and 8)

Fay_WuH_*DUSP19*_rs10200193: Fay-Wu's H scores for all the three populations (CEU, CHB and YRI) in the 3'UTR surrounding the SNP rs10200193 in *DUSP19* gene. It shows a strong dip (H< -20; highlighted in pink) in ~200kb region around this SNP in the CHB population.

Supplementary table S7: It contains data from 1000 Genomes for integrated Haplotype Score (iHS) and Derived Allele Frequency (DAF) values for 1) CEU population, 2) CHB population, and 3) YRI population.

Functional role of the 31 genes containing SNPs that exhibit signatures of positive selection and population differentiation (global FST>0.3, iHS>2.0 in any of the three populations and high DAF).

Supplementary table S8: Conservation analysis across species: The conservation of Alu-miRNA target sites for the validated genes was checked in Human, Chimpanzee, Rhesus, Gorilla, Marmoset, Orangutan, Baboon and Mouse.

Supplementary table S9: PolymiRTS_High-Fst: The conservation of Alu-miRNA SNPs under selection and their potential to disrupt target sites have been extracted from PolymiRTS database. High-Fst_High-iHS: Information for Alu-miRNA SNPs selected on the basis of Fst (>0.3) and iHS (>2.0).

Supplementary Figure legends

Supplementary Figure S1. *A flow diagram outlining the steps (as well as the filtering criteria) involved in the experimental work-flow for validating Alu-miRNA interactions.*

Supplementary Figure S2. *Ectopic expression of miR-15a-3p at 4.8nM causes G1 arrest.*

Treatment with a higher dose of miR-15a-3p mimic (4.8nM) results in an increased G1 cell population, suggestive of a possible G1-S arrest. The trend is even higher in case of scrambled probe. The G2/M cell population is marginally higher in case of miR-15a-3p treatment compared to that of scrambled oligo.

Supplementary Figure S3. *Isoform specific targeting by Alu-miRNA interaction.*

A) UCSC genome view for *RAD1* show that Alu-miRNA target within *RAD1* is present in all expressed transcripts. By virtue of this, they can potentially impact the protein levels of *RAD1* differentially due to the presence of miR-15a-3p target within Alu.

B) Alternately, Alus can only be a part of the alternate transcript isoform for a particular gene. miRNA target for *UBE2I* is present only within one alternate transcript, while the longest isoform and other alternate isoforms don't harbor miR-302d-3p targets.

Supplementary Figure S4. *A flow diagram summarizing the analysis pipeline for positive selection on Alu-miRNA target sites.*

Supplementary Figure S5. *Enrichment of miRNA targets within Alu in the 3'UTR of Alu-exonized transcripts.*

miRNA target density was found to be significantly different between Alu exonized and non-exonized transcripts and also within Alus in the 3'UTRs of Alu exonized transcripts compared to their corresponding non-Alu regions **(S5a & S5b)**. In the 3'UTR of Alu exonized transcripts, both the SNP density as well as the density of high F_{ST} SNPs were significantly greater within Alus compared to their corresponding non-Alu regions **(S5c & S5d)**. Also SNP density as well as density of high F_{ST} SNPs were found to be significantly greater in canonical miRNA targets compared to Alu-miRNA sites. However, compared to the background Alu sequence, these were not significantly different **(S5e & S5f)**. All the graphs represent probability distribution functions

(PDF) of the specified parameters (miRNA target density, SNP density, etc.) in the 3'UTR of Alu exonized and/or non exonized transcripts. The Y axes in all these graphs represent the kernel density of the PDF.

Supplementary Figure S6. *Both high F_{ST} (global F_{ST}>0.3) as well as high iHS (>2.0) SNPs are overrepresented within the 3'UTR-resident exonized Alus compared to the corresponding non-Alu regions.* A comparative analysis of SNP distribution (SNPs with global F_{ST}>0.3 or SNPs with iHS>2 vs. total SNPs) between Alu and non-Alu regions of the 3'UTRs in CEU, CHB and YRI populations shows this difference to be significant (p=0.02382, Student's t-test).

Supplementary Figure S7. a.) *Fay-Wu's H score shows a strong dip (H=-33.18) in ~200kb region around a SNP (rs10200193) in the 3'UTR of DUSP19 gene in CHB population, indicative of positive selection.*

b.) *Fay-Wu's H score shows a strong dip (H=-62.00) in ~100kb region around the SNPs (rs10158065, rs11122049) in the 3'UTR of NOL9 gene in CEU population, indicative of positive selection. It is also very low (H=-42.24) in CHB population, but not so in YRI (H=-2.76).*

Supplementary Figure S8. *Alu-miRNA sites are enriched for high F_{ST} and iHS SNPs*

Both iHS (>2.0) as well as global F_{ST} (>0.3) SNPs show a non-random enrichment within Alu-miRNA target sites of exonized transcripts across all the three populations (YRI, CEU and CHB).

Supplementary Figure S9. *Conservation of Alu-miRNA targets across primates at the DNA level*

There is a patchy conservation and a lot of sequence level mismatches (indicated by the red vertical lines) even for the organisms in which a BLAT search against the human *RAD1* gene sequence yielded result. Alu-miRNA sites were either altogether absent or occurred in isolated cases.

Supplementary Figure S10. *50% of the genes which contain 3'UTR SNPs with multiple signatures of positive selection within their Alu-miRNA target sites, form a tightly-connected network centered on the UBC gene.*

This network contains significantly more interactions than what would be expected for a random set of proteins of similar size, drawn from the whole genome. Such enrichment indicates that the proteins are at least partially biologically connected, as a group. Edges represent protein-protein interactions and are marked by lines. Line colour indicates the type of interaction evidence available in STRING version: 10.0 (magenta: experimentally determined, light green: text mining). Interaction score cut-off used ≥0.4 (medium confidence).

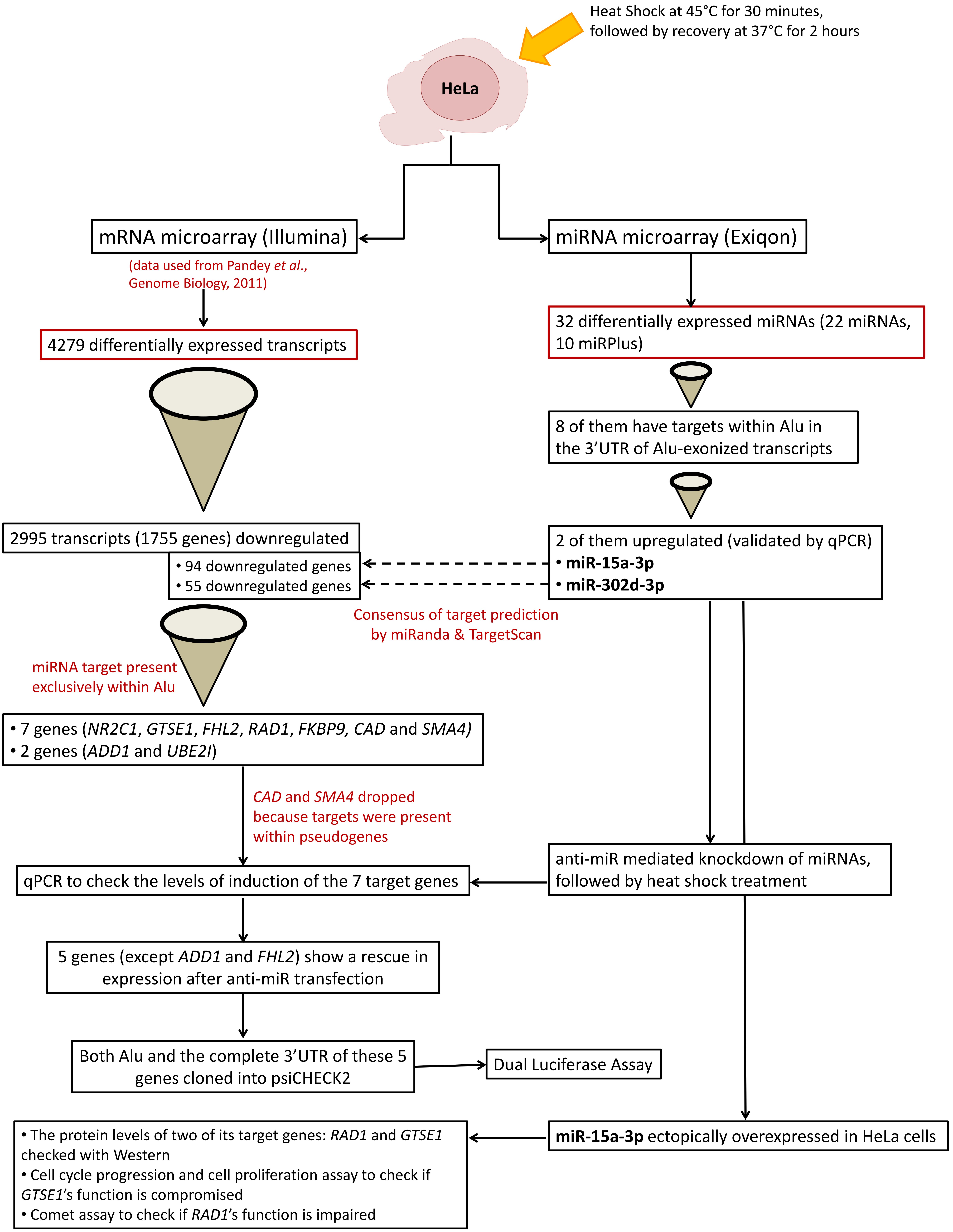

Supplementary Figure S1

Cell cycle progression Assay

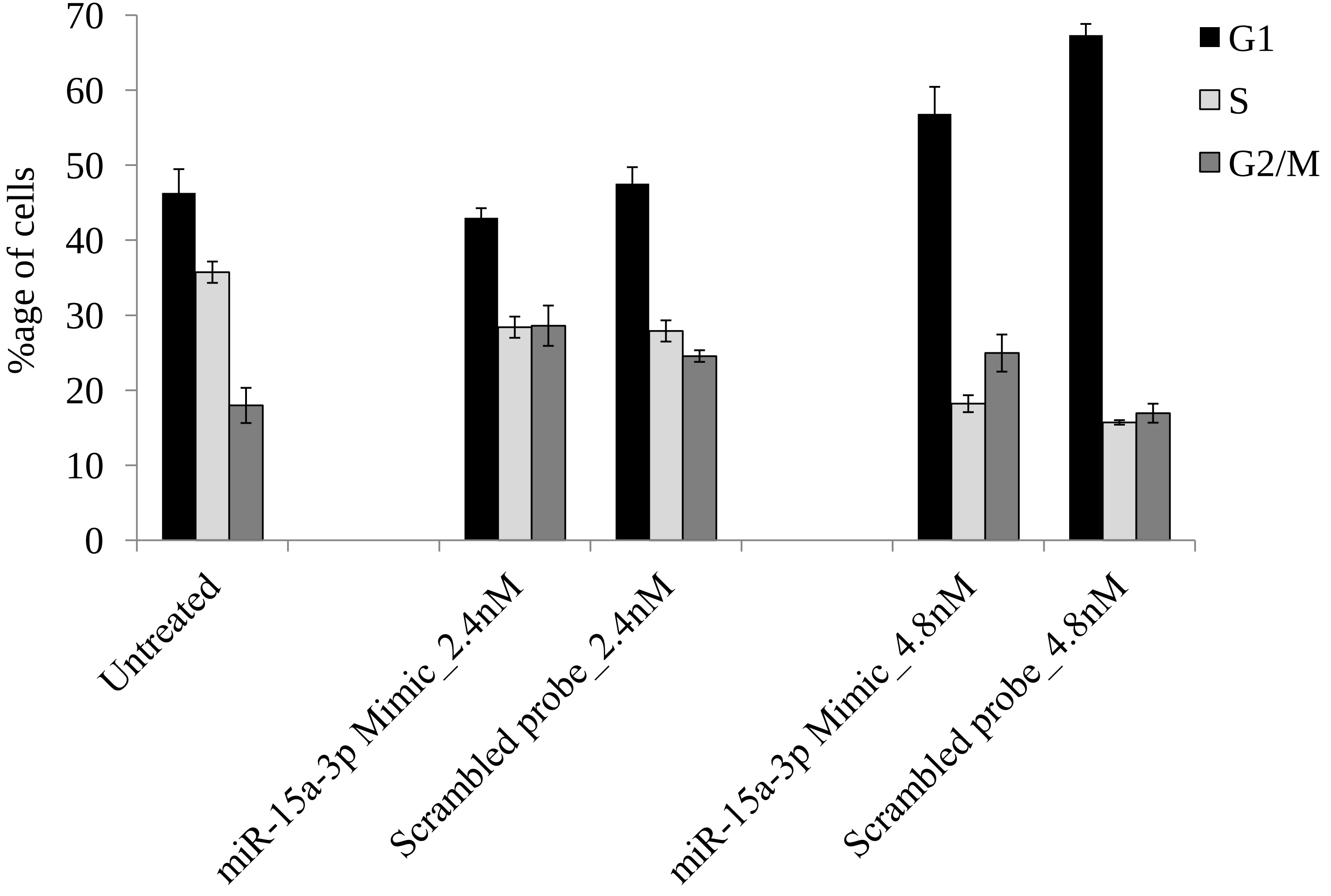

Supplementary Figure S2

A. **miRNA target within Alu is present in all transcript isoforms**

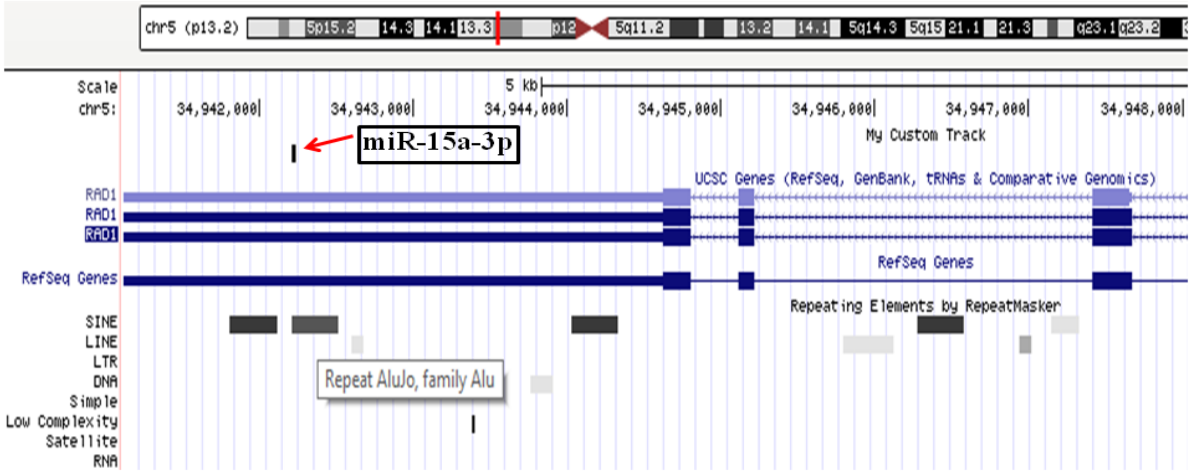

B. **miRNA target within Alu is present only in alternate isoform**

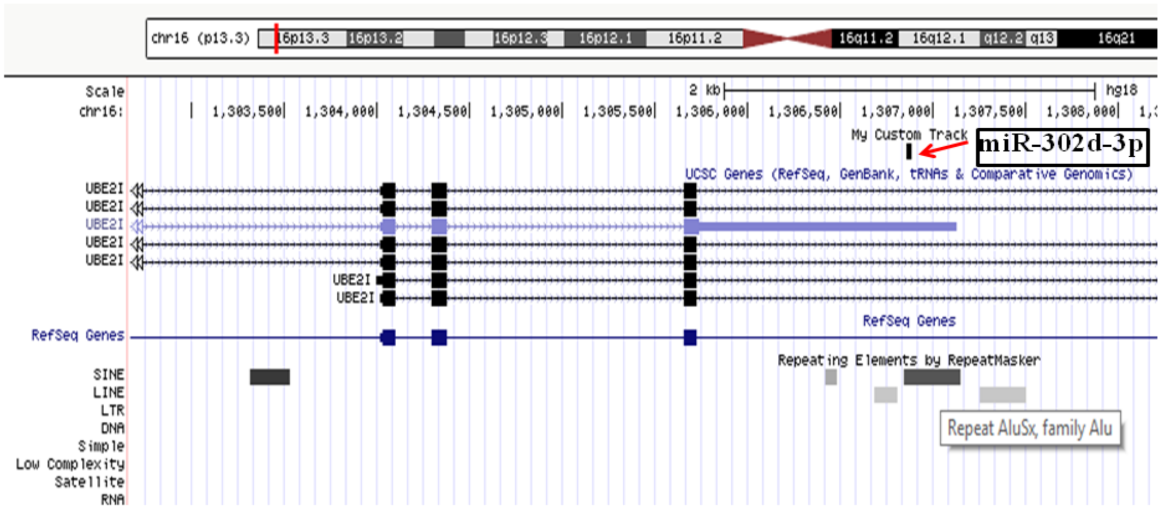

Supplementary Figure S3

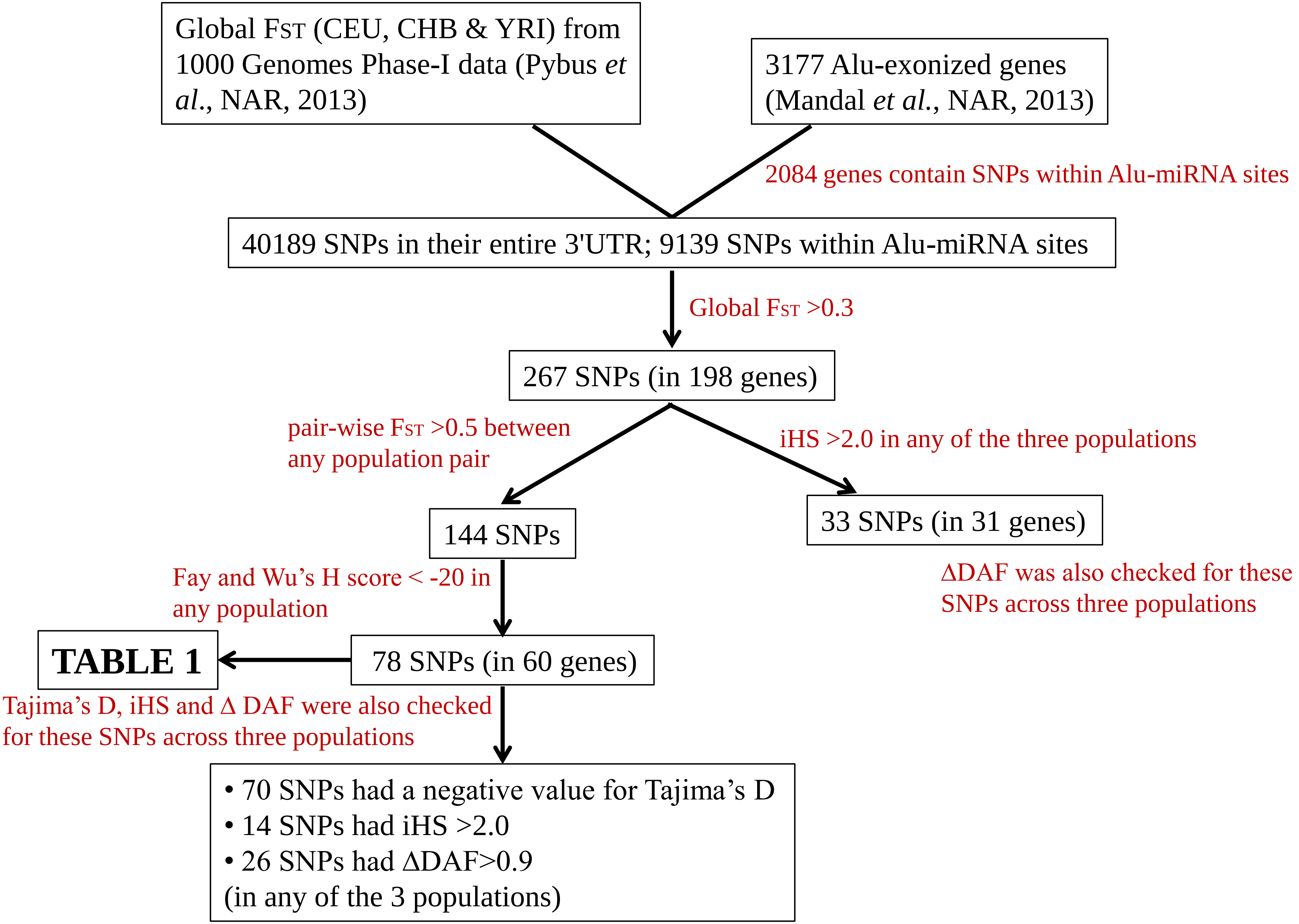

Supplementary Figure S4

miRNA target density in the 3'UTR of Alu exonized and non-Alu exonized transcripts

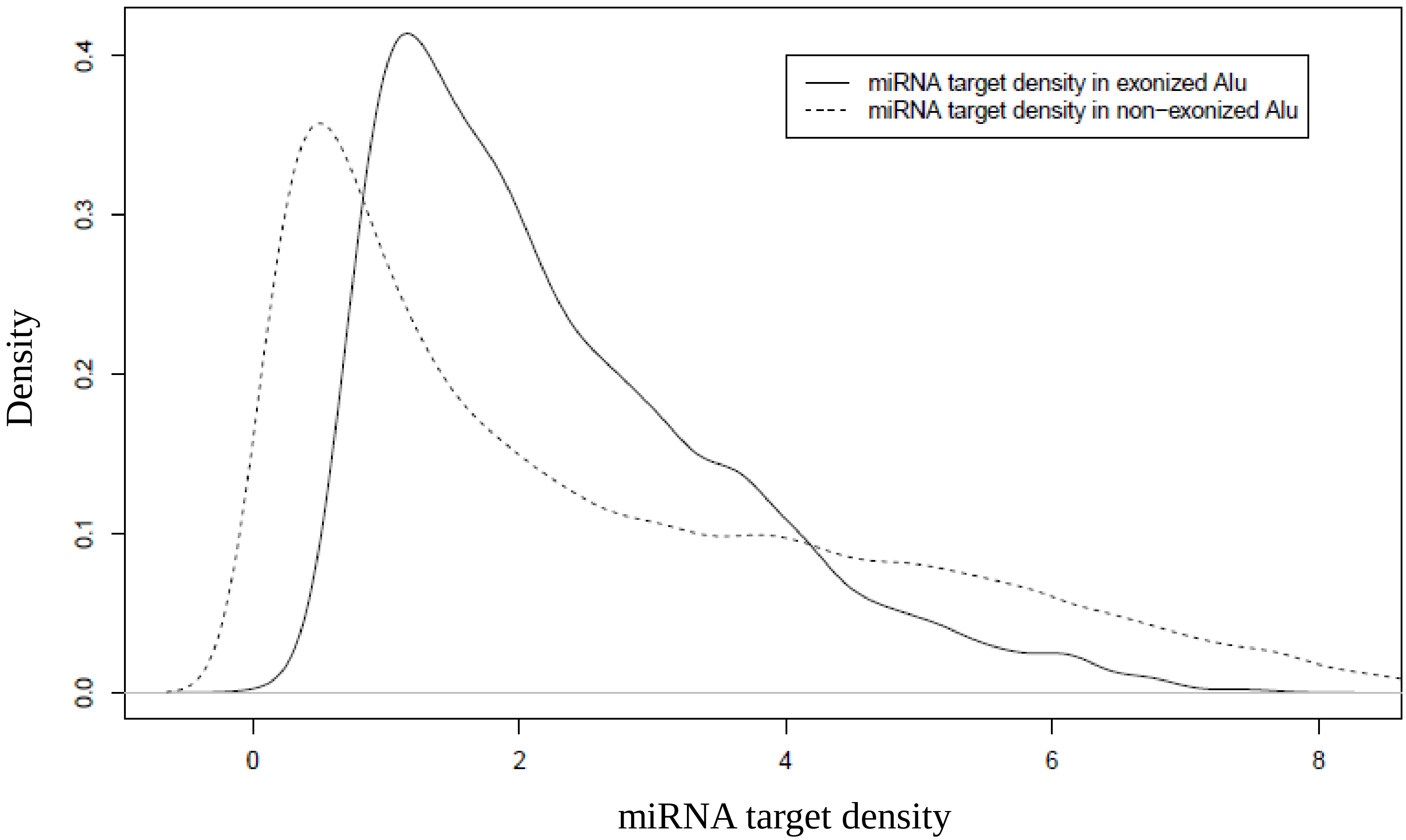

Supplementary Figure S5a

miRNA target density in the 3'UTR within Alu and non-Alu regions of Alu exonized transcripts

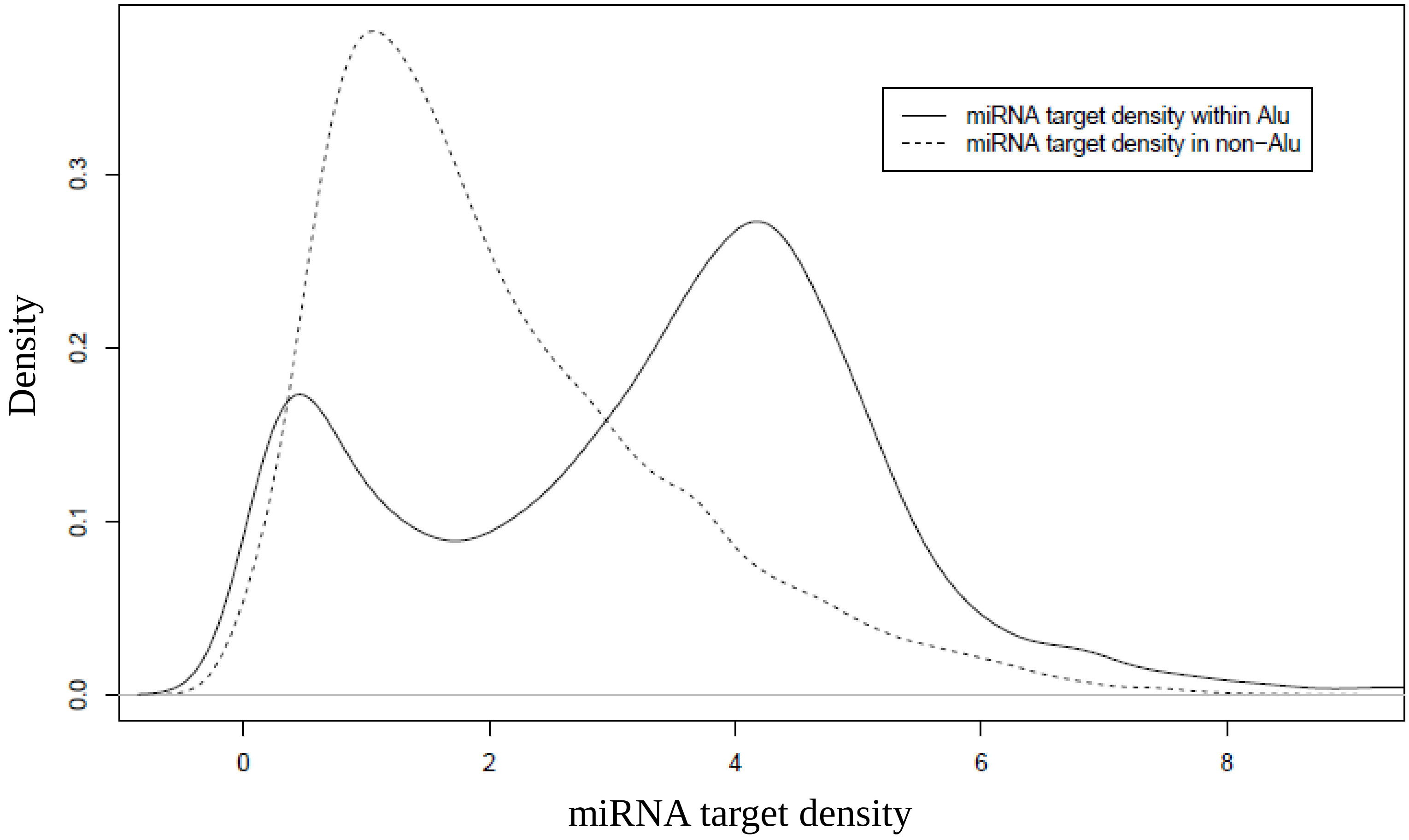

Supplementary Figure S5b

SNP density within Alu and non-Alu regions of 3'UTR of Alu exonized transcripts

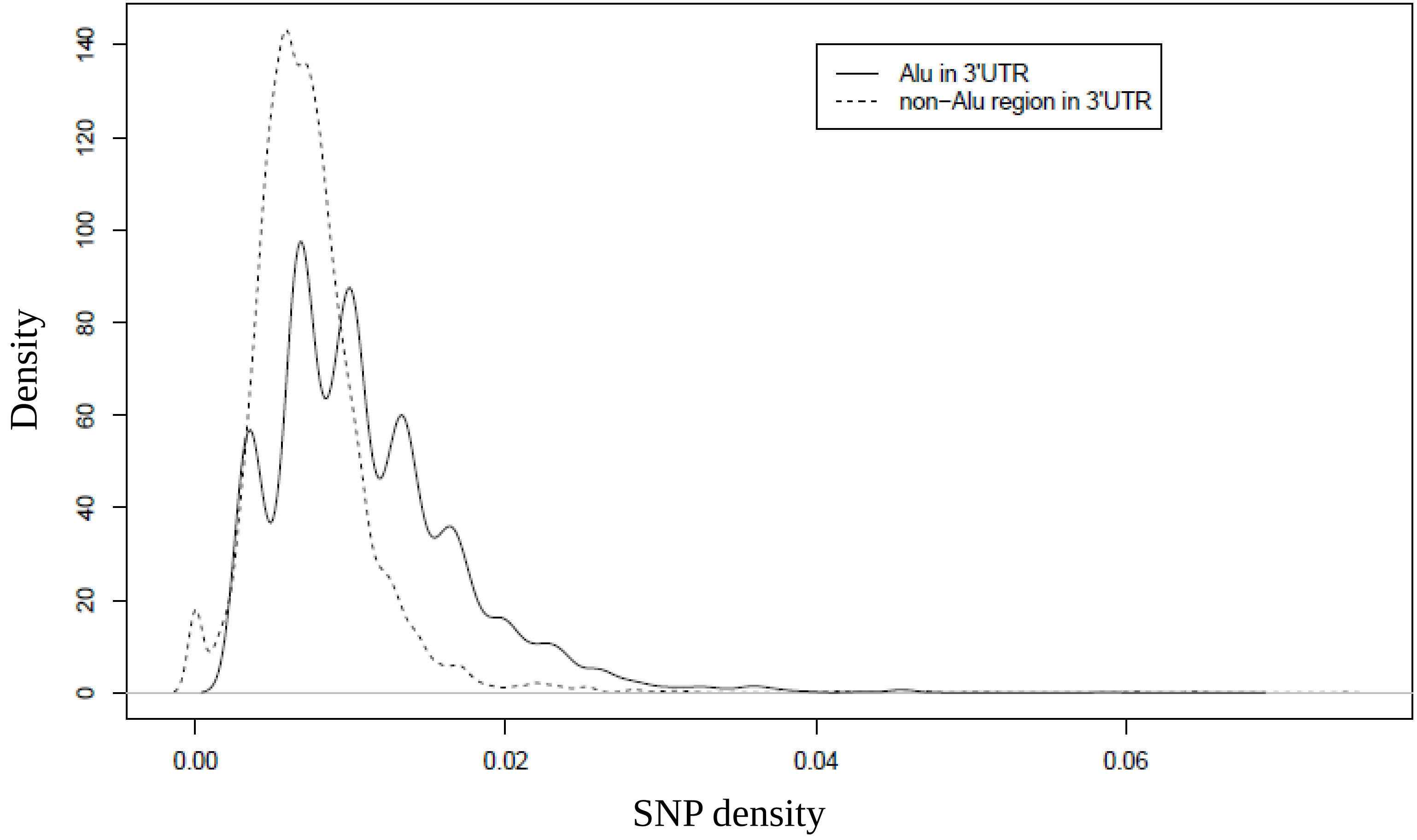

Supplementary Figure S5c

High global FST SNP density within Alu and non-Alu regions of 3'UTR of Alu exonized transcripts

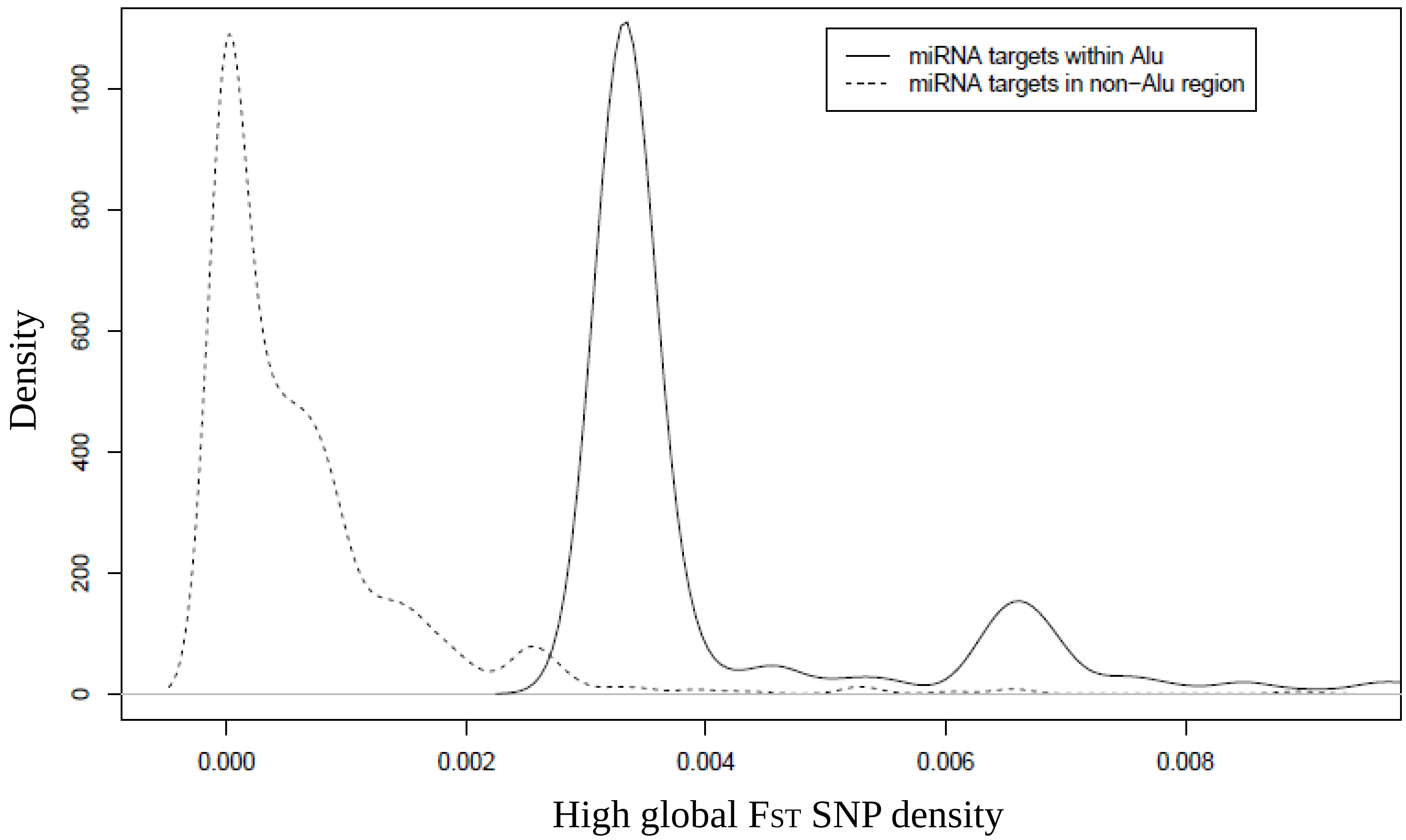

Supplementary Figure S5d

Distribution of total SNPs in 3'UTR of Alu exonized transcripts

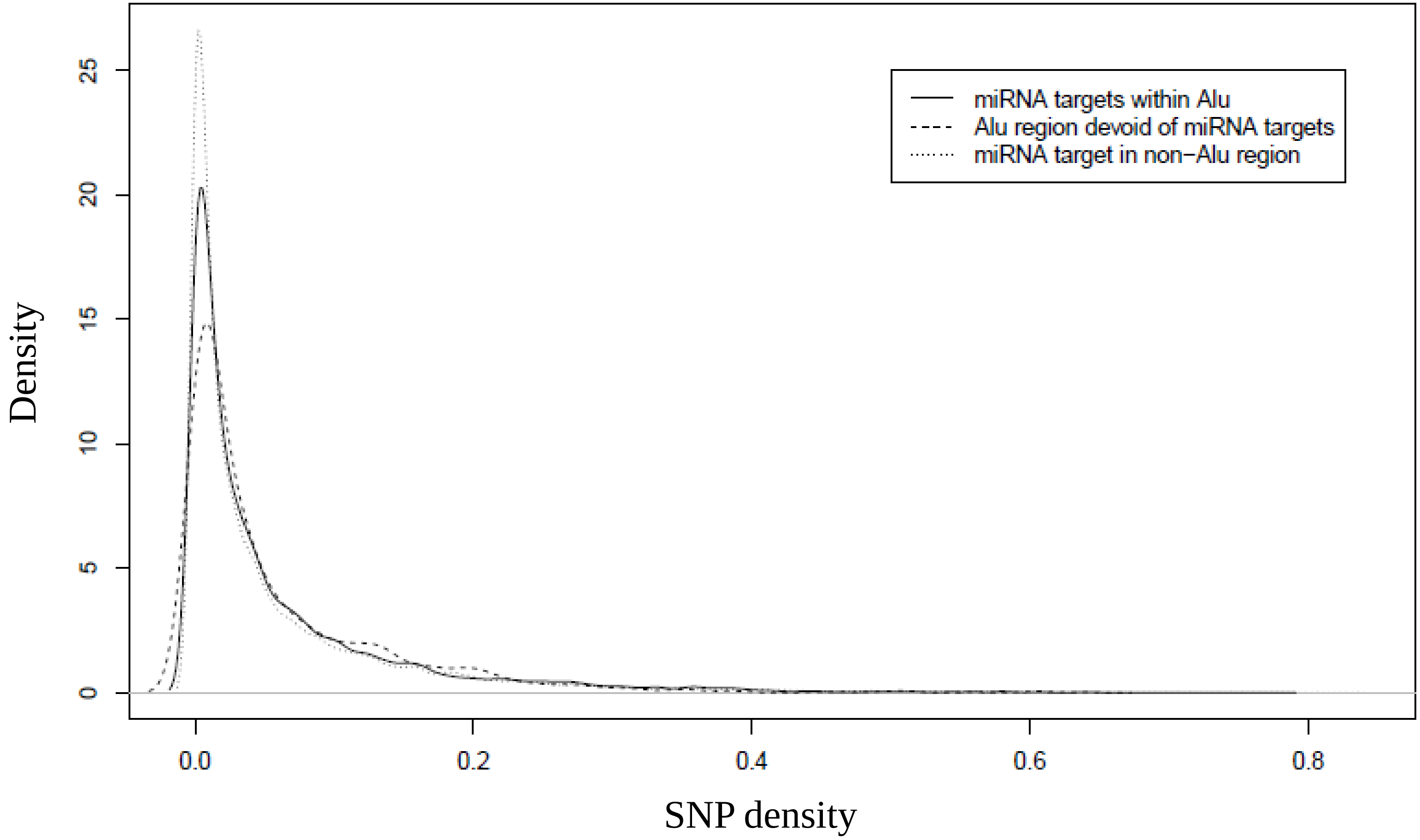

Supplementary Figure S5e

Distribution of high global F_{ST} SNPs in 3'UTR of Alu exonized transcripts

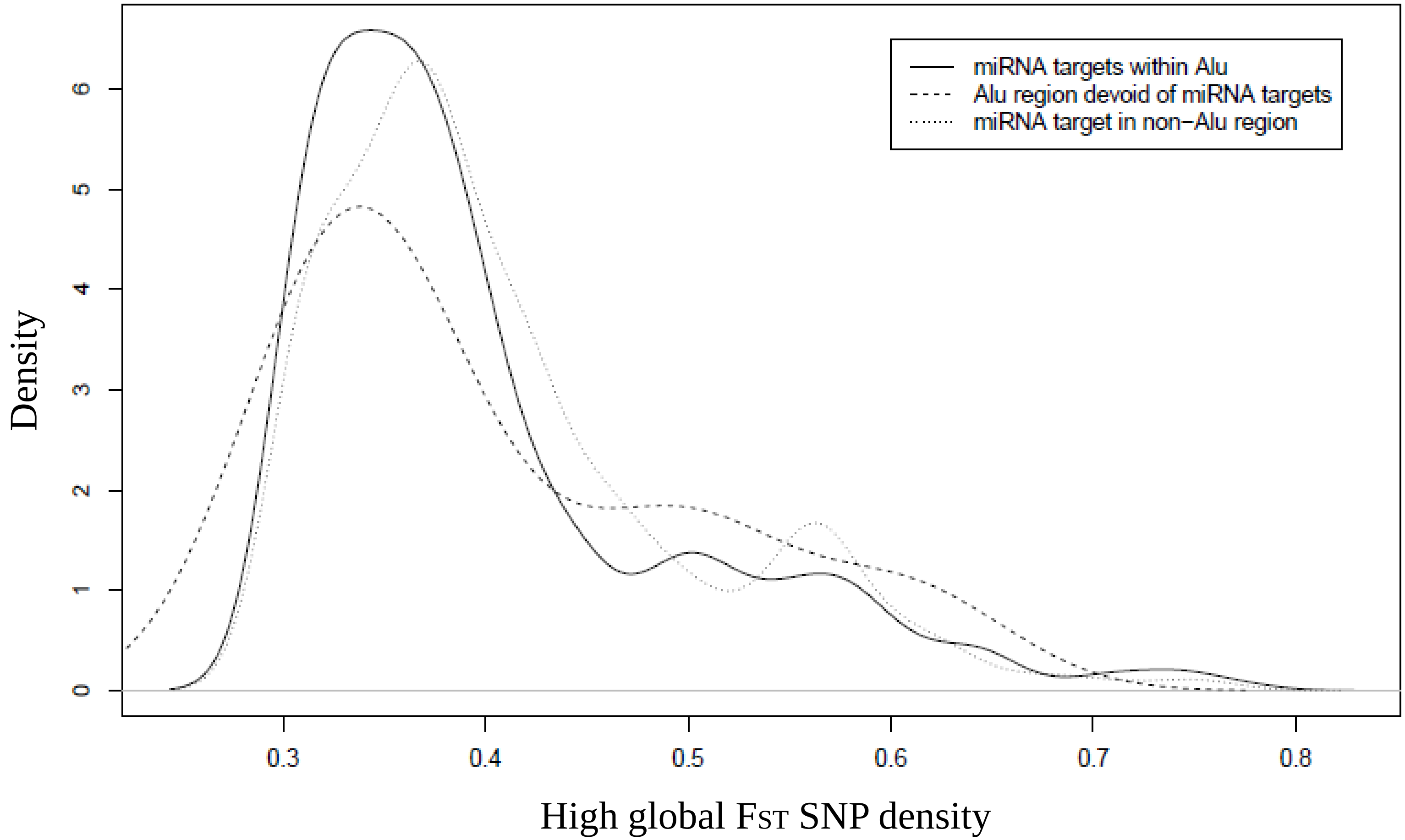

Supplementary Figure S5f

High global F_{ST} (>0.3) SNPs vs. total SNPs in Alu and non-Alu regions of 3'UTR

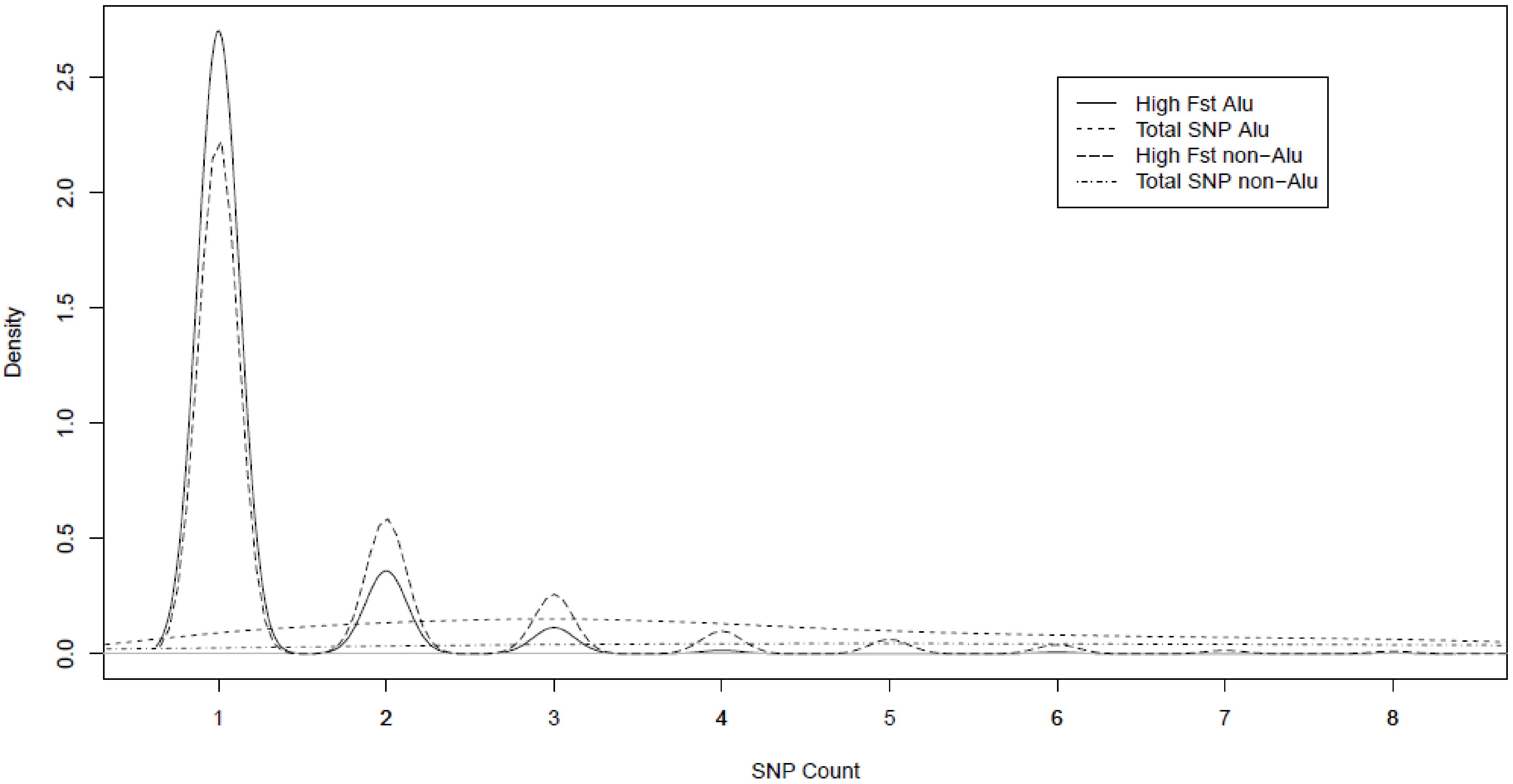

A comparative analysis of SNP distribution (SNPs with global F_{ST}>0.3 vs. all SNPs) in both Alu and non-Alu regions of the 3'UTRs in (CEU + CHB + YRI) populations.

Supplementary Figure S6a

High iHS (>2.0) SNPs vs. total SNPs in Alu and non-Alu regions of 3'UTR

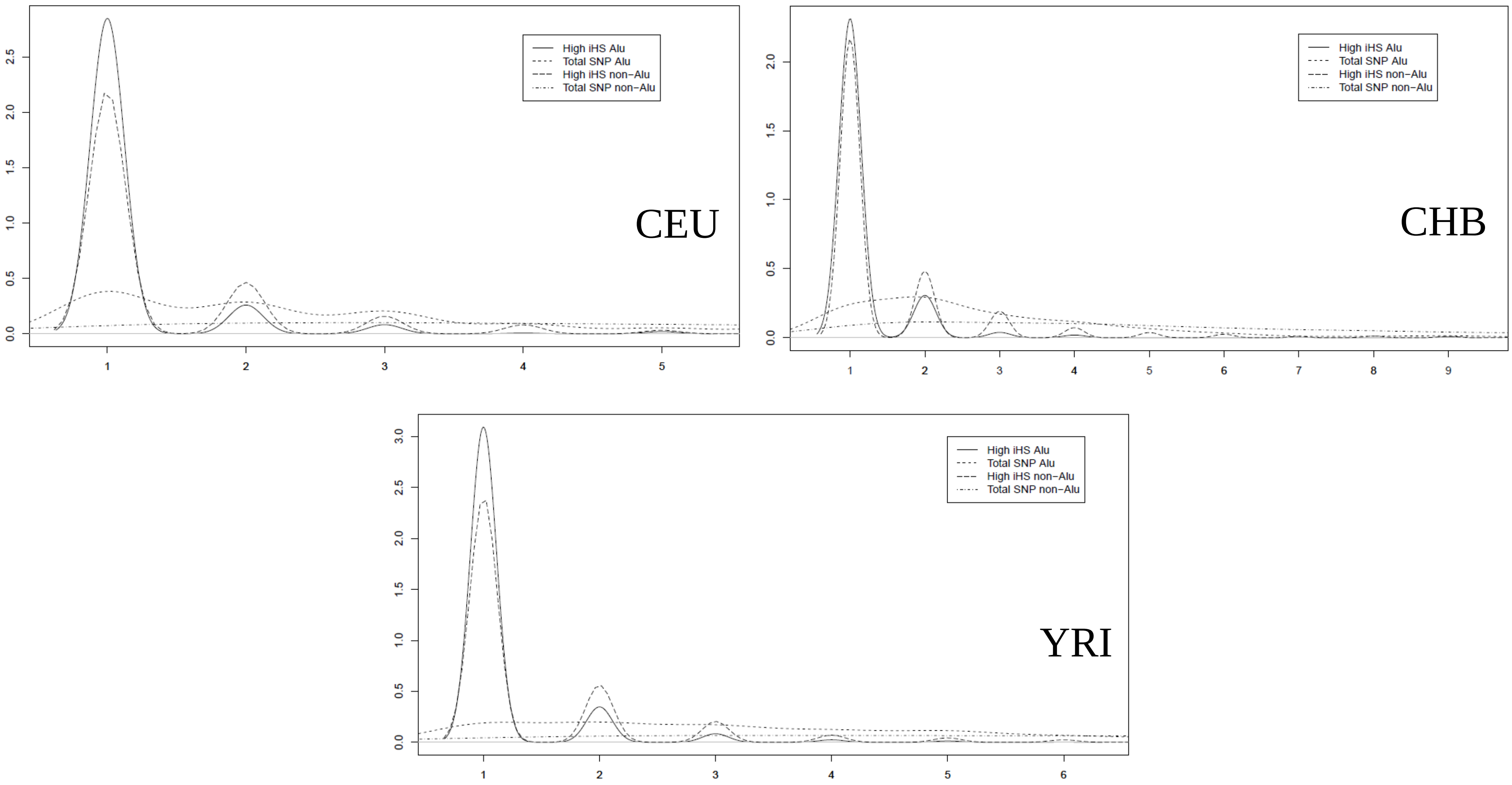

A comparative analysis of SNP distribution (SNPs with iHS>2.0 vs. all SNPs) in both Alu and non-Alu regions of the 3'UTRs in CEU, CHB and YRI populations. X axis: SNP count, Y axis: kernal density of the probability distribution function.

Supplementary Figure S6b

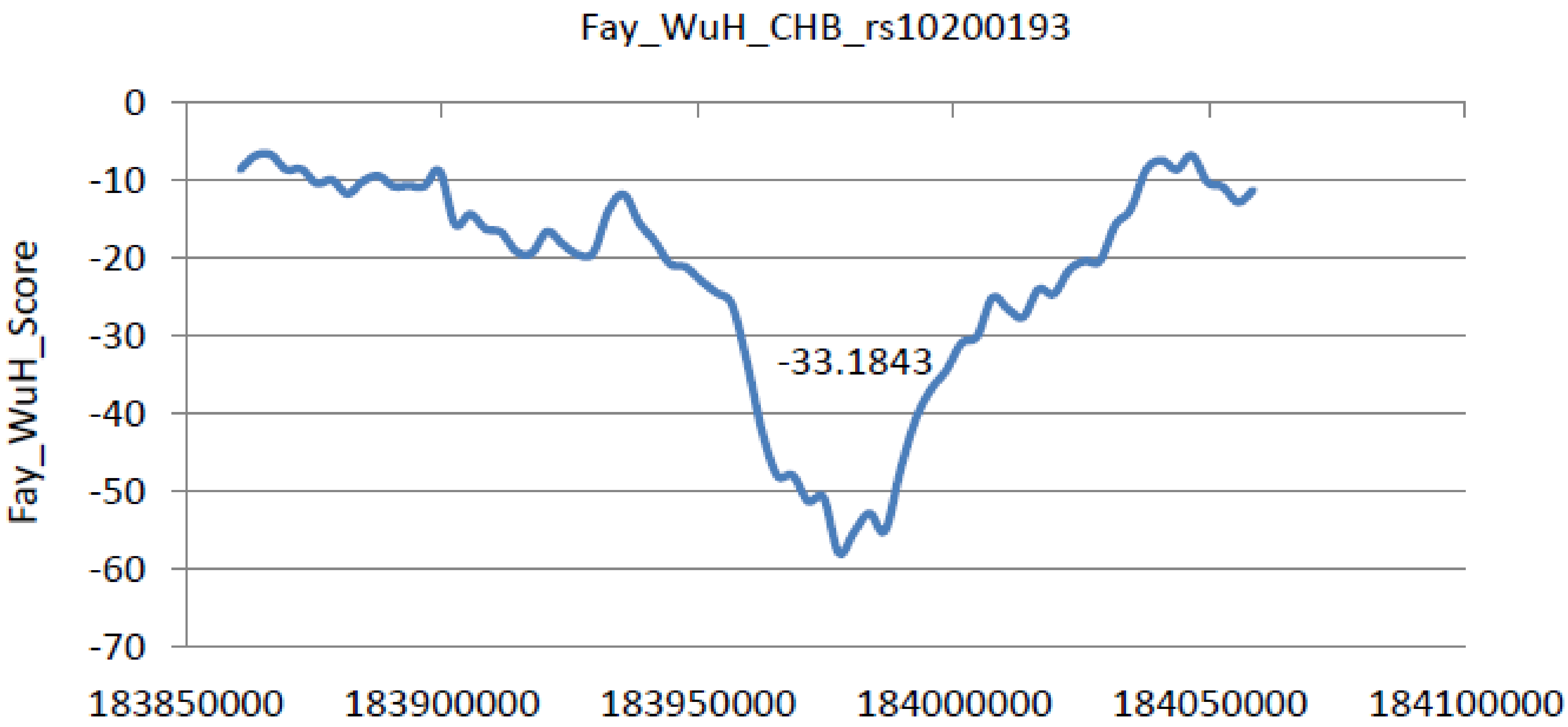

Fay-Wu’s H score shows a strong dip (H=-33.18) in ~200kb region around a SNP (rs10200193) in the 3'UTR of *DUSP19* gene in CHB population, indicative of positive selection.

Supplementary Figure S7a

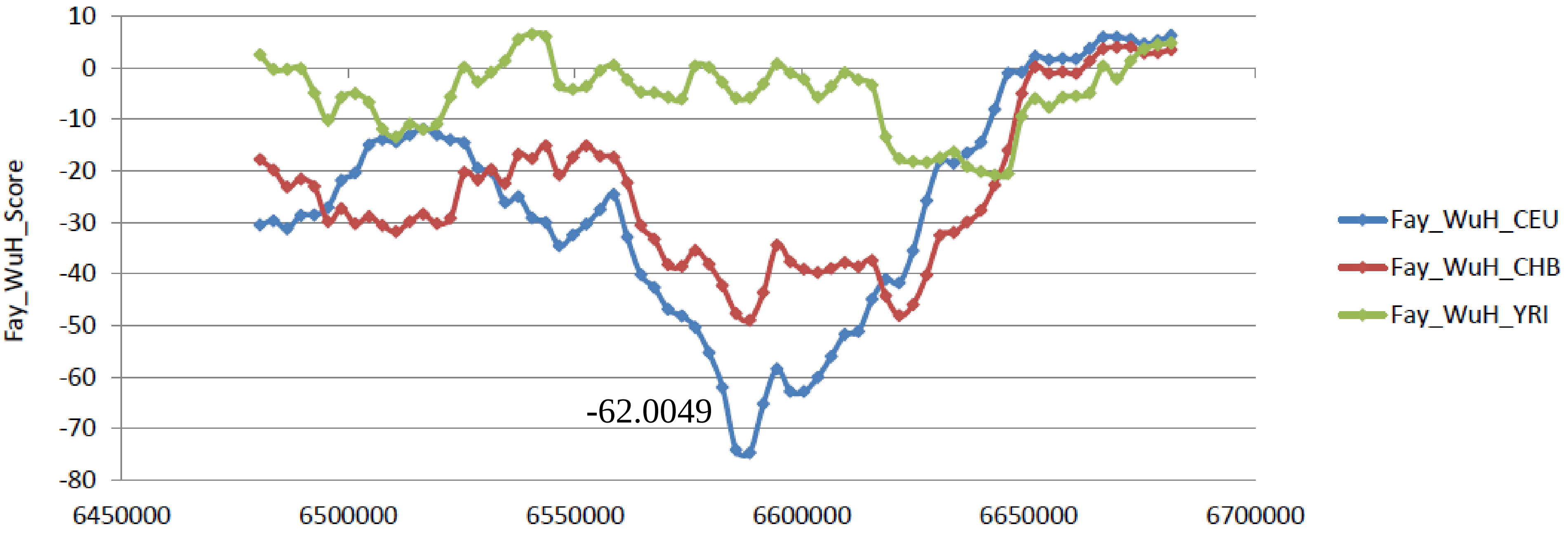

Fay-Wu's H score shows a strong dip (H= -62.00) in ~100kb region around the SNPs (rs10158065, rs11122049) in the 3'UTR of *NOL9* gene in CEU population, indicative of positive selection. It is also very low (H= -42.24) in CHB population, but not so in YRI (H= -2.76).

Supplementary Figure S7b

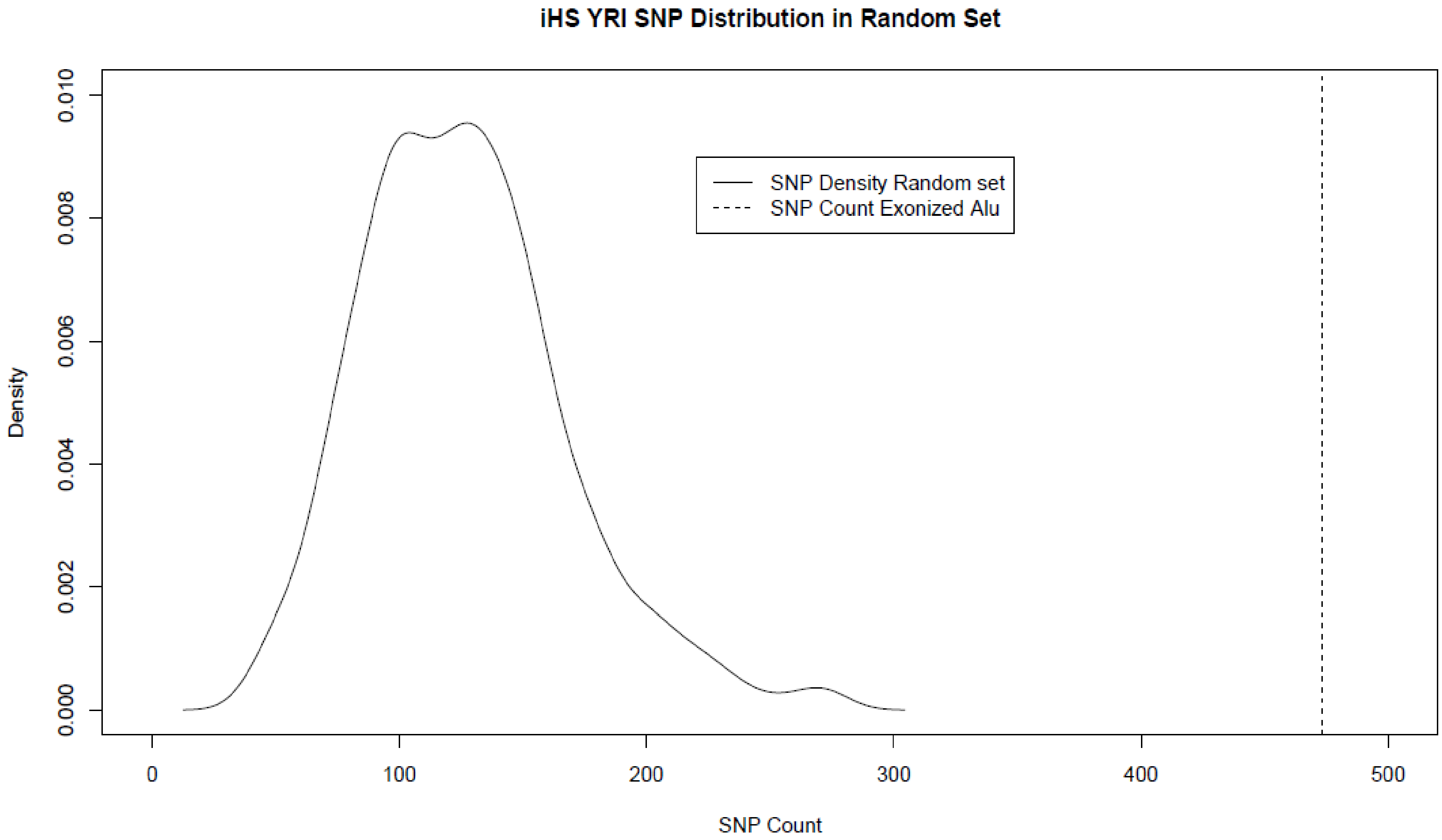

Density plot of iHS_YRI for all SNPs from 1000 random sets and SNP count in Alu-exonized 3'UTR. The plot shows that iHS for SNPs in exonized Alu are more than mean + 3*SD in 1000 random sets (~30 genes).

Mean= 124.59, SD=40.27, mean+ 3*SD =245.42, No. of SNP in YRI population = 473

Supplementary Figure S8a

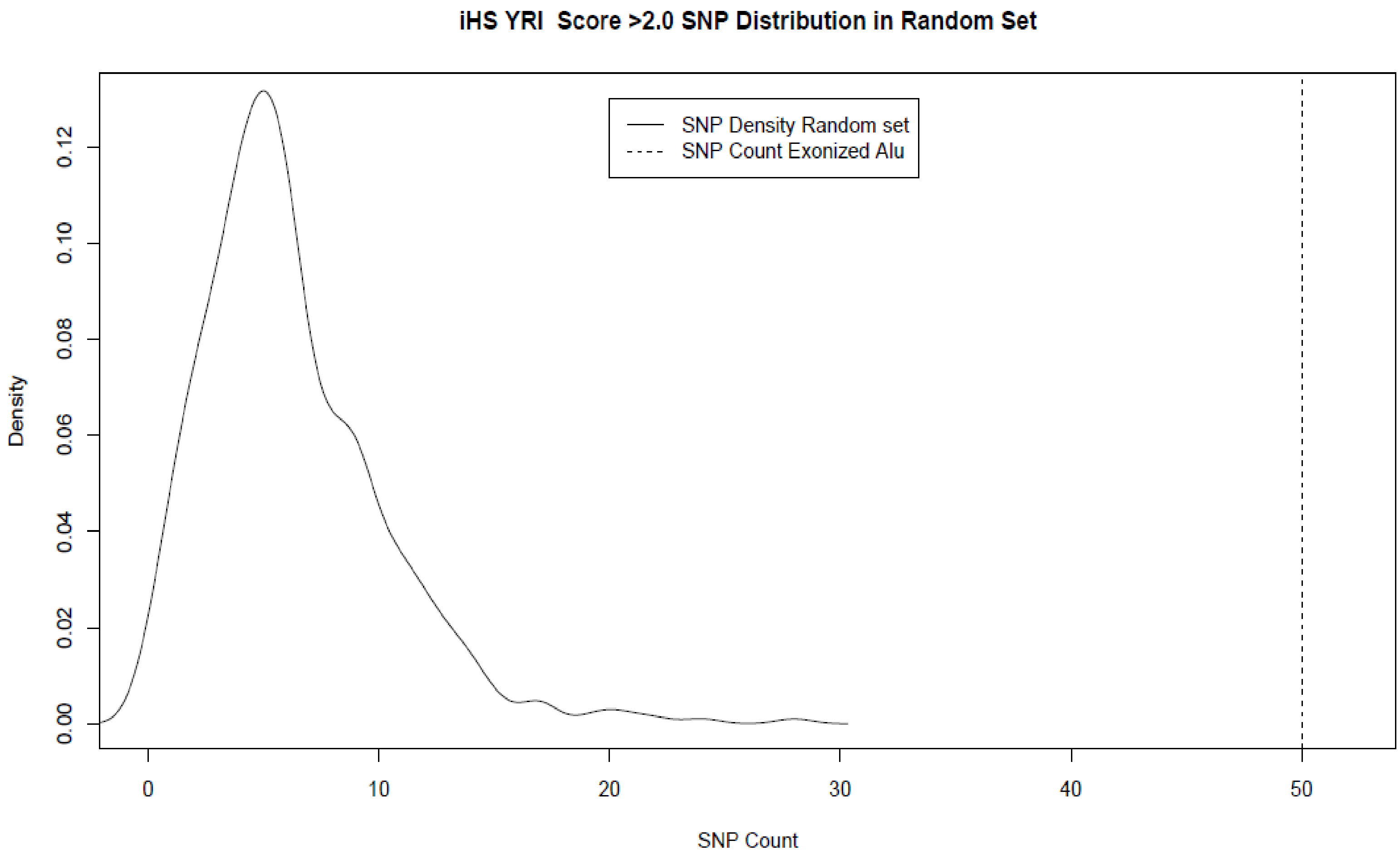

Density plot of iHS_YRI SNP (>2.0) from 1000 random sets and SNP count in Alu-exonized 3'UTR. The plot shows that iHS SNP in exonized Alu are more than mean + 3*SD in 1000 random sets (~30 genes).
Mean= 6.28, SD= 4.002, mean+ 3*SD = 18.29, No. of SNP in YRI population = 50

Supplementary Figure S8b

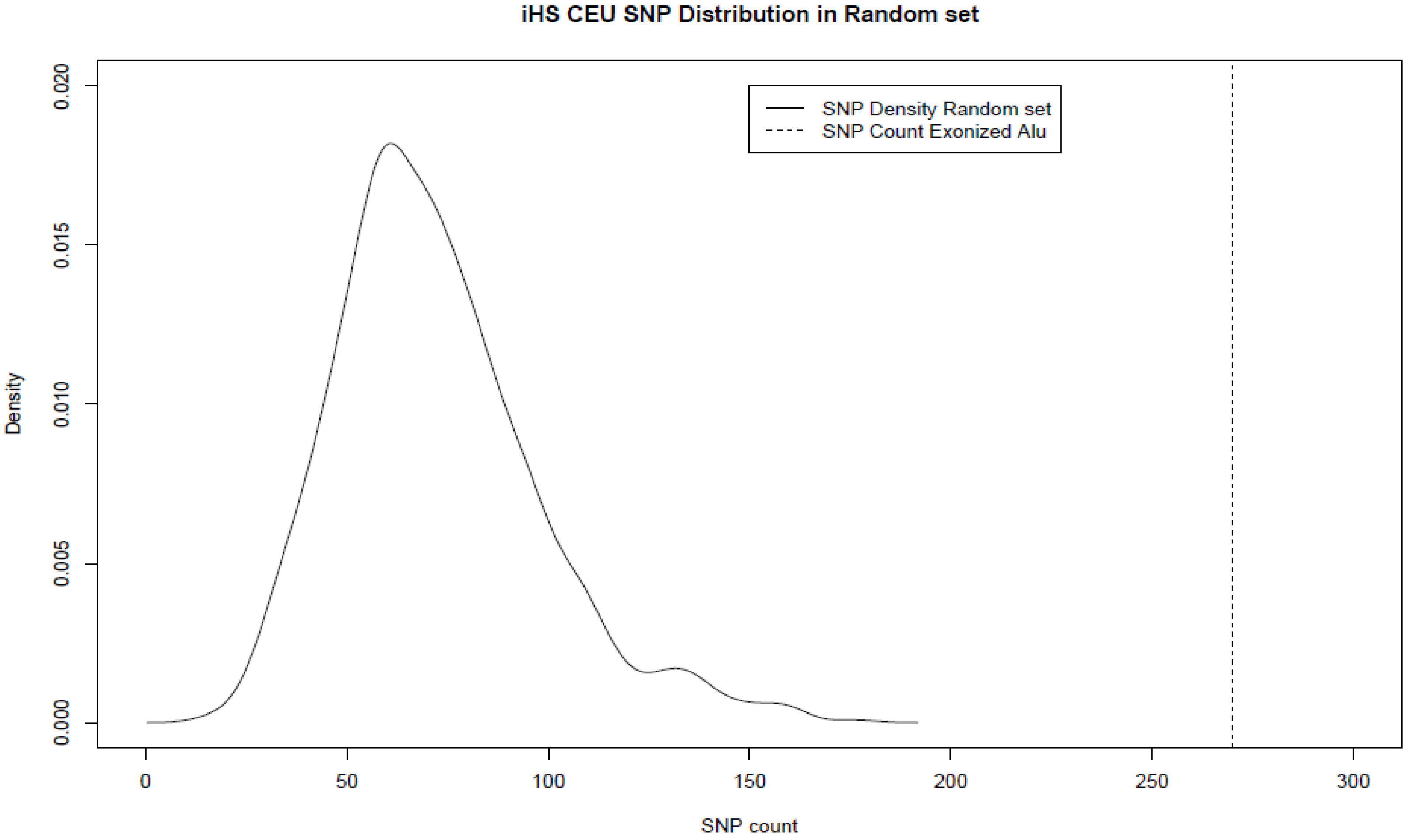

Density plot of iHS_CEU for all SNPs from 1000 random sets and SNP count in Alu-exonized 3’UTR. The plot shows that iHS for SNP in exonized Alu are more than mean + 3*SD in 1000 random sets (~30 genes).

Mean= 72.03, SD= 25.01, mean+ 3*SD = 147.06, No. of SNP in CEU population = 270

Supplementary Figure S8c

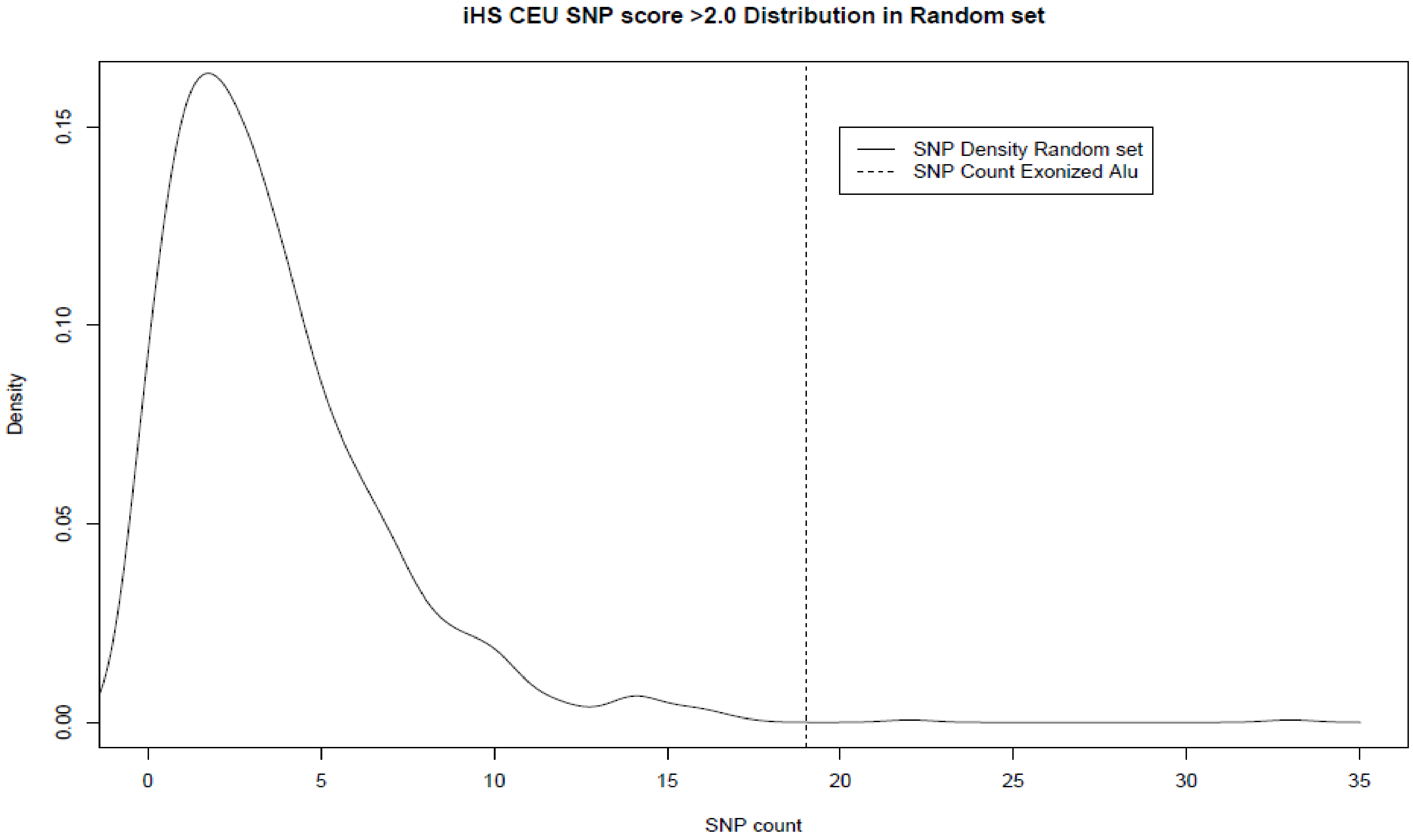

Density plot of iHS_CEU SNP (>2.0) from 1000 random sets and SNP count in Alu-exonized 3'UTR. The plot shows that iHS SNP in exonized Alu are more than mean + 3*SD in 1000 random sets (~30 genes).

Mean= 3.67, SD = 3.24, mean + 3*SD = 13.40, No. of SNP in CEU population = 19

Supplementary Figure S8d

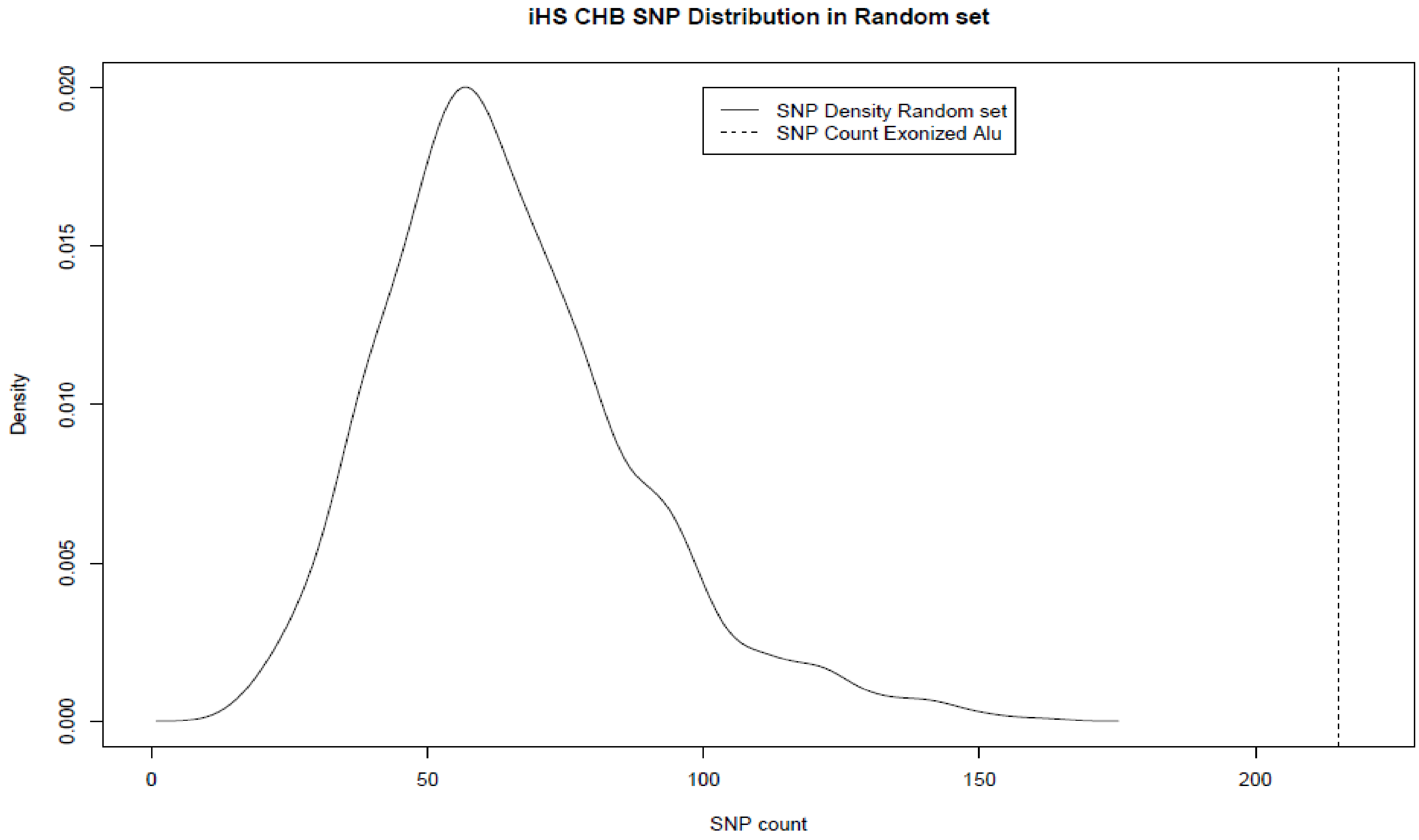

Density plot of iHS_CHB for all SNPs from 1000 random set and SNP count in Alu-exonized 3'UTR. The plot shows that iHS for SNPs in exonized Alu are more than mean + 3*SD in 1000 random sets (~30 genes).

Mean = 64.92, SD = 23.20, mean+ 3*SD = 134.54, No. of SNP in CHB population = 215

Supplementary Figure S8e

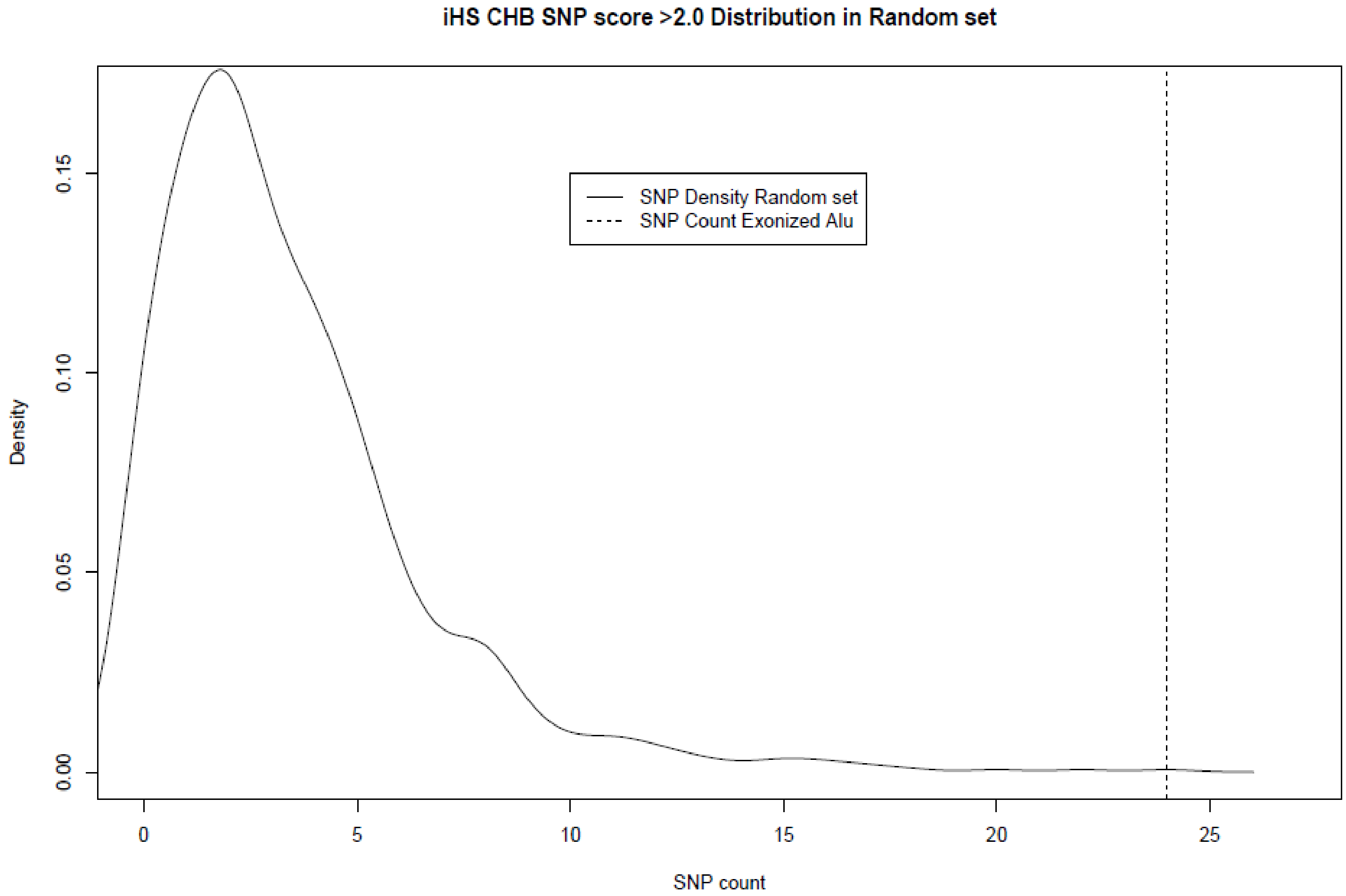

Density plot of iHS_CHB SNP (>2.0) from 1000 random sets and SNP count in Alu-exonized 3'UTR. The plot shows that iHS SNP in exonized Alu are more than mean + 3*SD in 1000 random sets (~30 genes).

Mean= 3.41, SD = 3.11, mean + 3*SD = 12.76, No. of SNP in CHB population = 24

Supplementary Figure S8f

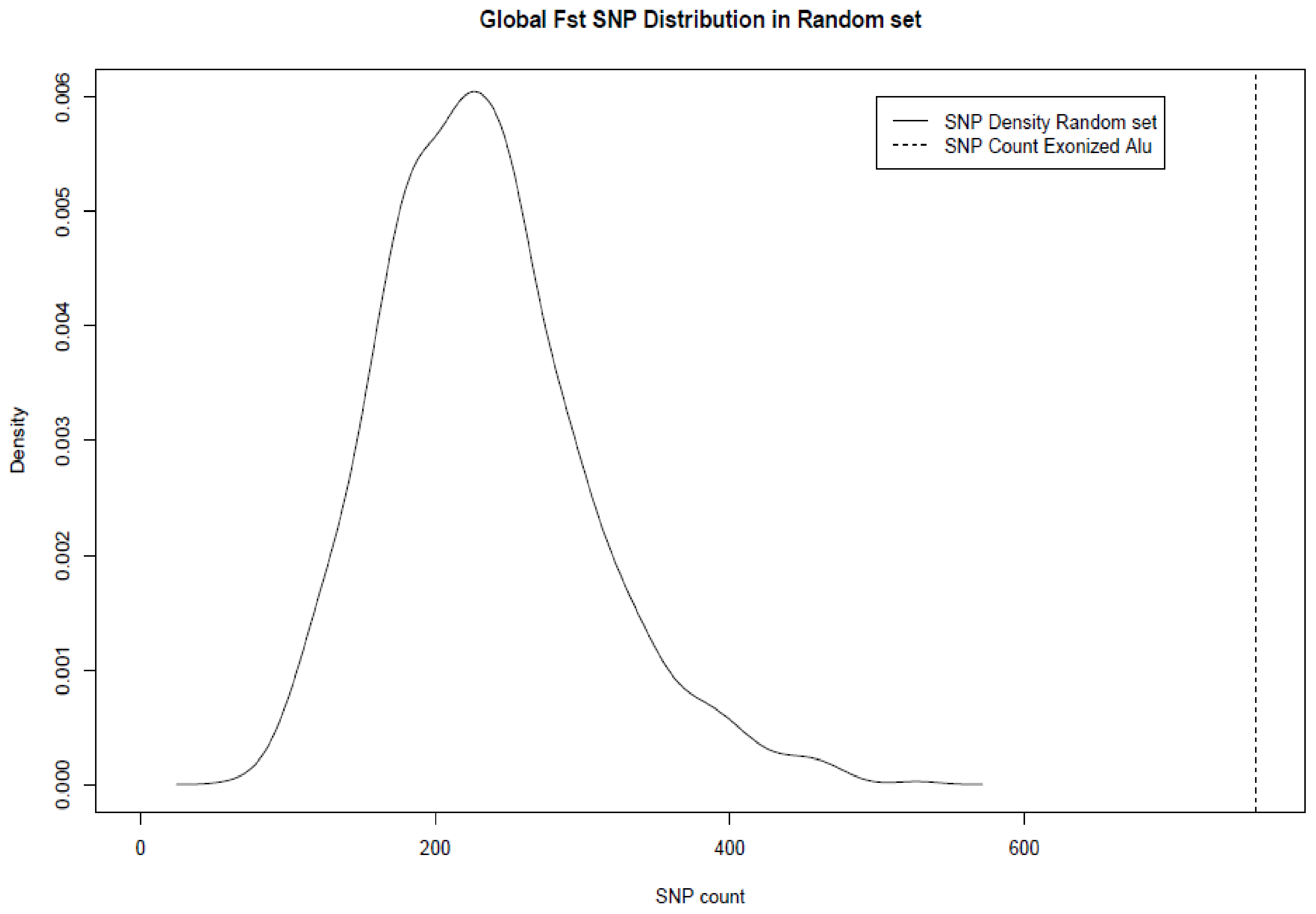

Density plot of global F_{ST} SNP from 1000 random sets and SNP count in Alu-exonized 3'UTR. The plot shows that iHS SNP in exonized Alu are more than mean + 3* SD in 1000 random sets (~30 genes).

Mean = 233.3, SD = 69.91, mean + 3*SD = 443.03, No. of SNP in global F_{ST} = 757

Supplementary Figure S8g

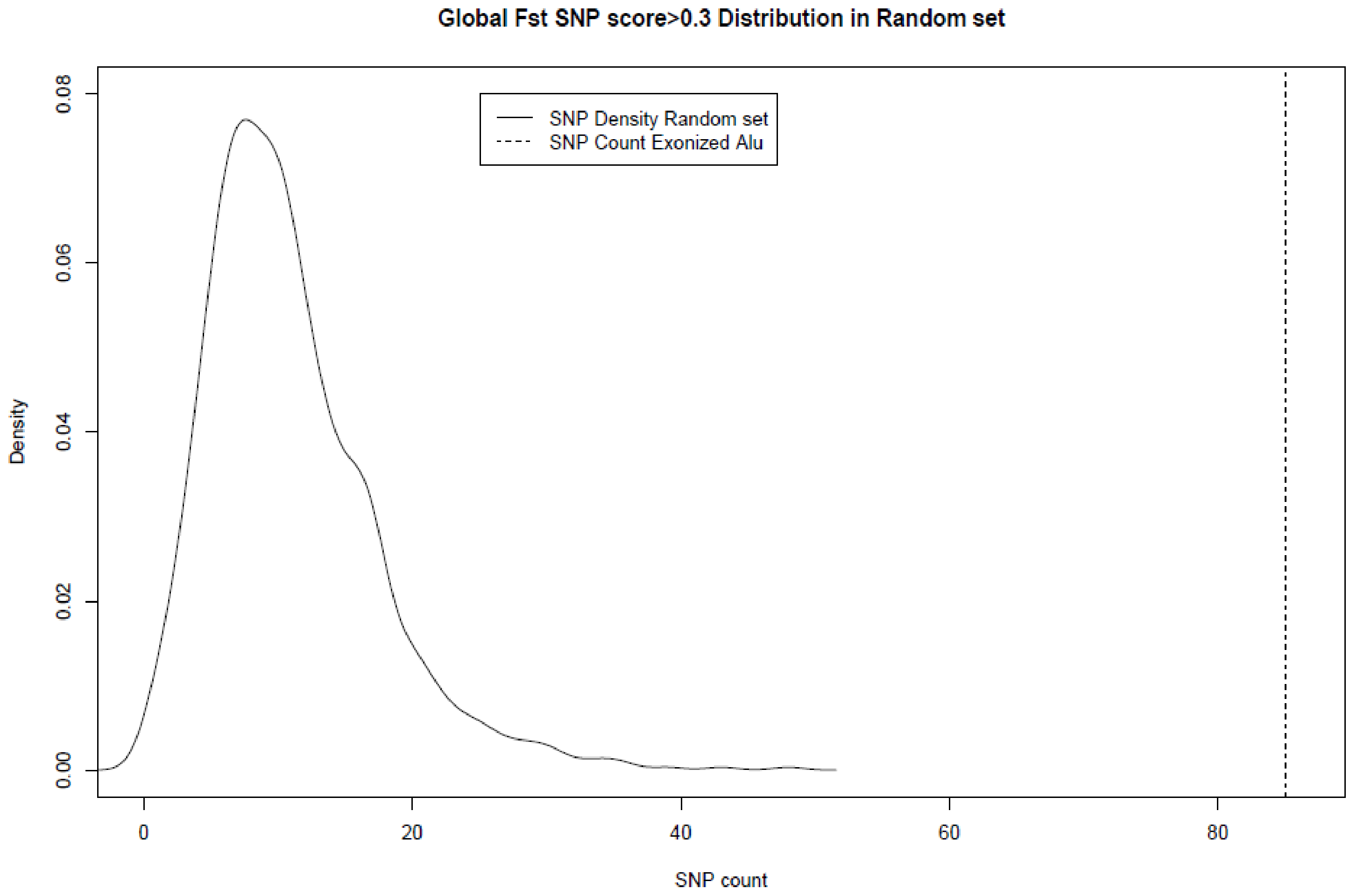

Density plot of global F_{ST} SNP (>0.3) from 1000 random sets and SNP count in Alu-exonized 3'UTR. The plot shows that F_{ST} SNP in exonized Alu are more than mean + 3*SD in 1000 random sets (~30 genes).
Mean= 10.88, SD = 6.26, mean + 3*SD = 29.68, No. of SNP in global F_{ST} = 85

Supplementary Figure S8h

Conservation of Alu-miRNA targets across Primates at DNA level

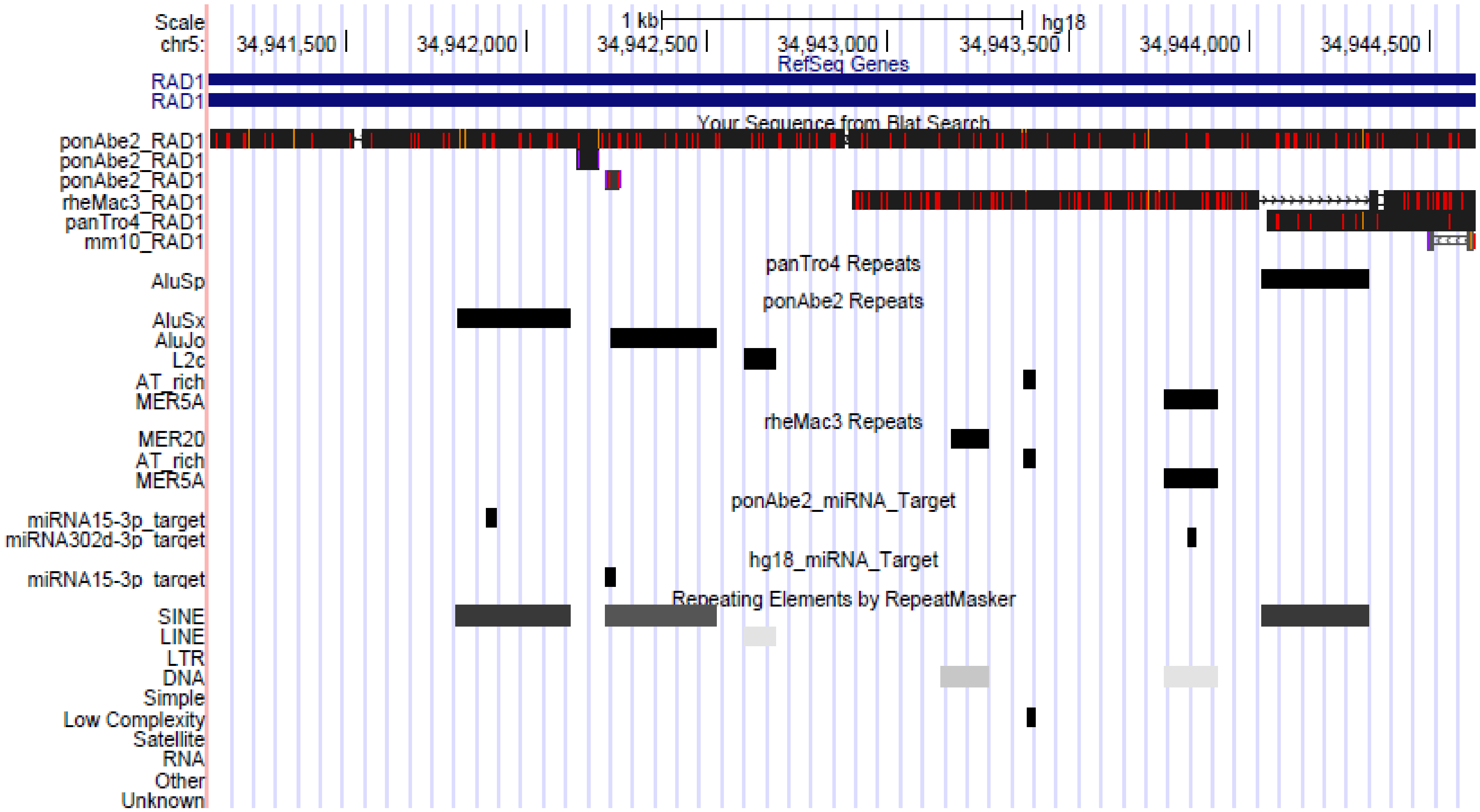

Supplementary Figure S9

50% of the genes which contain 3'UTR SNPs with multiple signatures of positive selection within their Alu-miRNA target sites, form a tightly connected network centered on *UBC*.

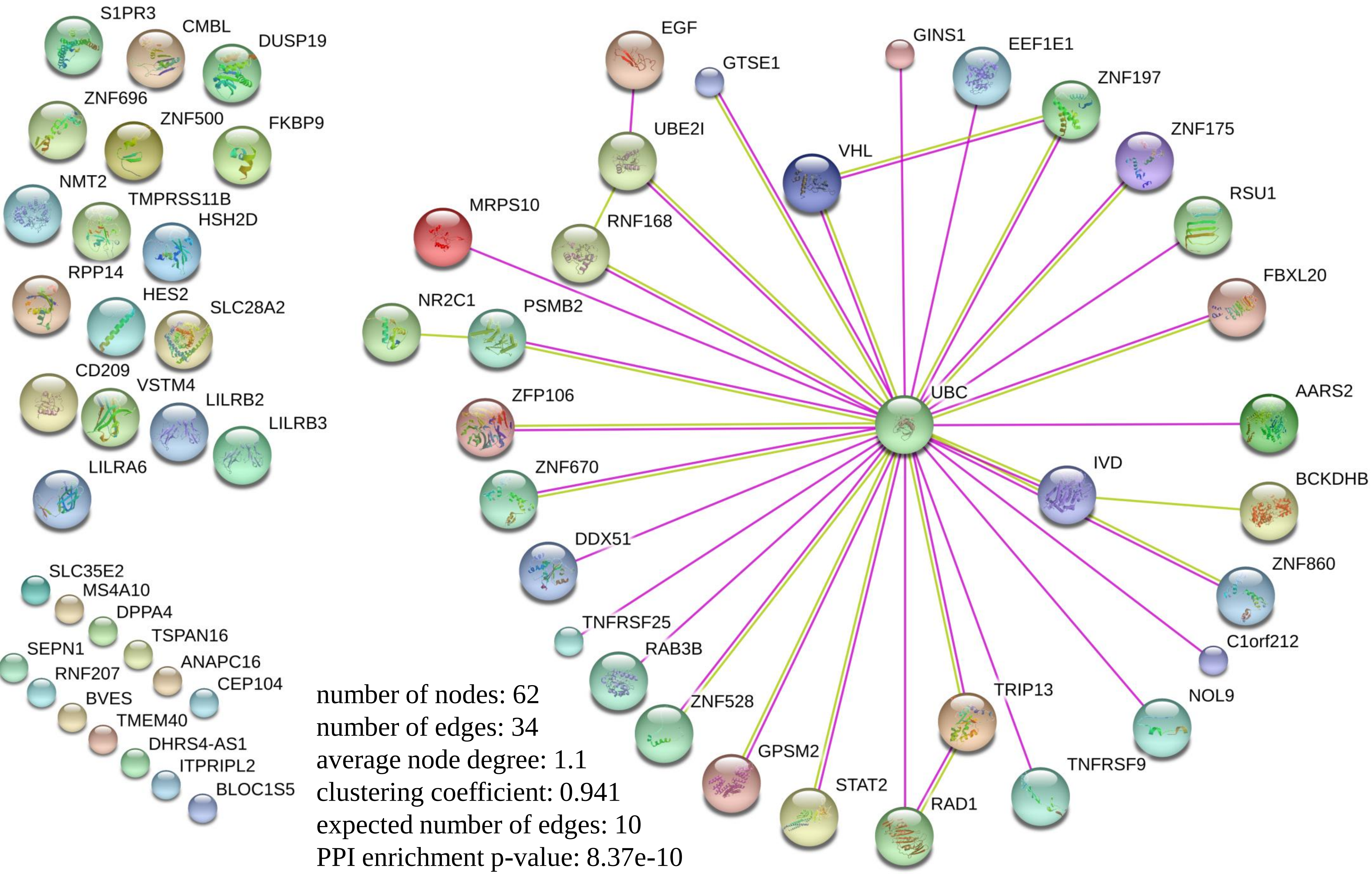

number of nodes: 62
number of edges: 34
average node degree: 1.1
clustering coefficient: 0.941
expected number of edges: 10
PPI enrichment p-value: 8.37e-10

Supplementary Figure S10
